# Hypermethylation of Hepatic Mitochondrial *ND6* Provokes Systemic Insulin Resistance

**DOI:** 10.1002/advs.202004507

**Published:** 2021-05-02

**Authors:** Ke Cao, Weiqiang Lv, Xueqiang Wang, Shanshan Dong, Xuyun Liu, Tielin Yang, Jie Xu, Mengqi Zeng, Xuan Zou, Daina Zhao, Qingqing Ma, Mu Lin, Jiangang Long, Weijin Zang, Feng Gao, Zhihui Feng, Jiankang Liu

**Affiliations:** ^1^ Center for Mitochondrial Biology and Medicine The Key Laboratory of Biomedical Information Engineering of Ministry of Education School of Life Science and Technology Xi'an Jiaotong University Xi'an Shaanxi 710049 China; ^2^ Frontier Institute of Science and Technology Xi'an Jiaotong University Xi'an Shaanxi 710049 China; ^3^ National & Local Joint Engineering Research Center of Biodiagnosis and Biotherapy The Second Affiliated Hospital of Xi'an Jiaotong University Xi'an Shannxi 710004 China; ^4^ Biomedical Informatics & Genomics Center The Key Laboratory of Biomedical Information Engineering of Ministry of Education School of Life Science and Technology Xi'an Jiaotong University Xi'an Shannxi 710049 China; ^5^ Guizhou Aerospace Hospital Zunyi Guizhou 563099 China; ^6^ Department of Pharmacology School of Basic Medical Sciences Xi'an Jiaotong University Health Science Center Xi'an Shaanxi 710061 China; ^7^ School of Aerospace Medicine Fourth Military Medical University Xi'an 710032 China

**Keywords:** DNA methyltransferase 1 (DNMT1), insulin resistance, mitochondrial dysfunction, mitochondrial NADH‐dehydrogenase 6 (ND6), obesity and type 2 diabetes mellitus (T2DM)

## Abstract

Mitochondrial epigenetics is rising as intriguing notion for its potential involvement in aging and diseases, while the details remain largely unexplored. Here it is shown that among the 13 mitochondrial DNA (mtDNA) encoded genes, NADH‐dehydrogenase 6 (*ND6*) transcript is primarily decreased in obese and type 2 diabetes populations, which negatively correlates with its distinctive hypermethylation. Hepatic mtDNA sequencing in mice unveils that *ND6* presents the highest methylation level, which dramatically increases under diabetic condition due to enhanced mitochondrial translocation of DNA methyltransferase 1 (DNMT1) promoted by free fatty acid through adenosine 5’‐monophosphate (AMP)‐activated protein kinase (AMPK) activation. Hepatic knockdown of *ND6* or overexpression of *Dnmt1* similarly impairs mitochondrial function and induces systemic insulin resistance both in vivo and in vitro. Genetic or chemical targeting hepatic DNMT1 shows significant benefits against insulin resistance associated metabolic disorders. These findings highlight the pivotal role of *ND6* epigenetic network in regulating mitochondrial function and onset of insulin resistance, shedding light on potential preventive and therapeutic strategies of insulin resistance and related metabolic disorders from a perspective of mitochondrial epigenetics.

## Graphical abstract

Metabolic disorders emerge as one of the biggest public health challenges with distinctive feature of elevated free fatty acid, which can promote mitochondrial translocation of DNA methyltransferase 1 in liver, resulting in hypermethylation of NADHdehydrogenase 6 (ND6) on mitochondrial DNA. Such molecular change suppresses ND6 expression and induces hepatic mitochondrial dysfunction, eventually provokes systemic insulin resistance.

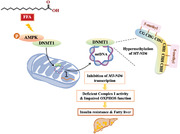

## 1 Introduction

Insulin resistance (IR) is a predominant feature of type 2 diabetes mellitus (T2DM) with obesity as one of the key risk factors. Being the major organ involved in glucose metabolism, the liver plays a central role in the development of IR.^[^ ^1^ ^]^ Recent studies by us ^[^ ^2^, ^3^ ^]^ and others ^[^ ^4^, ^5^ ^]^ have revealed a strong correlation between impaired mitochondrial oxidative phosphorylation (OXPHOS) capacity and IR. A deficiency of mitochondrial OXPHOS could result in both a decrease in ATP production and a profound increase in reactive oxygen species (ROS) production, eventually leading to an imbalance in energy metabolism and hepatic IR.^[^ ^4^ ^]^ Targeting mitochondrial OXPHOS has been considered a preventive and therapeutic approach against hepatic IR and T2DM.

The integrity of mitochondria is dynamically regulated by both nuclear DNA (nDNA) and mitochondrial DNA (mtDNA), the latter only encodes 13 proteins that present a minority of the respiratory chain subunits. Nevertheless, they are indispensable for maintaining mitochondrial OXPHOS function.^[^ ^6^ ^]^ As the only protein encoded by mtDNA L‐strand, mitochondrial NADH‐dehydrogenase 6 (ND6) has been demonstrated to be the key component of complex I for its critical role in the proper assembly of complex I.^[^ ^7^ ^]^ Mutation of *ND6* causes severe mitochondrial respiratory dysfunction ^[^ ^8^ ^]^ and mitochondrial genetic diseases, mainly including Leber hereditary optic neuropathy and Leigh syndrome.^[^ ^9^ ^]^ Recently, a clinical meta‐analysis identified two variants of *ND6* associated with body mass index (BMI) in obese subjects.^[^ ^10^ ^]^ Previous studies also showed a reduction of *ND6* transcription in the adipose tissue of mice fed a high fat diet (HFD) ^[^ ^11^ ^]^ and in non‐alcoholic fatty liver.^[^ ^12^ ^]^ These findings suggest a potential involvement of *ND6* in the development of metabolic disorders. However, no studies have fully characterized the detail mechanism of *ND6* associated regulatory network in the disease onset and intervention.

nDNA methylation is the best understood epigenetic process which has been demonstrated to modulate nDNA‐encoded mitochondrial gene transcription, thus participating in IR and T2DM,^[^ ^13^ ^]^ while mtDNA methylation has been largely overlooked and controversial since the 1970s.^[^ ^14^, ^15^, ^16^ ^]^ Despite several studies exhibited the absence of mtDNA methylation mainly based on the relatively low level of CpG methylation compared with nDNA,^[^ ^17^, ^18^, ^19^, ^20^ ^]^ a series of investigations have revealed the methylation of mtDNA in both CpG ^[^ ^12^, ^21^, ^22^, ^23^ ^]^ and non‐CpG ^[^ ^23^, ^24^ ^]^ patterns by using methylation‐specific PCR,^[^ ^12^ ^]^ antibody‐based methylated DNA immunoprecipitation ^[^ ^22^, ^23^ ^]^ or pyrosequencing and bisulfite sequencing.^[^ ^21^, ^23^ ^]^ The discovery of DNA methyltransferases (DNMTs) in mitochondria has provided substantial evidence for the presence of mtDNA methylation. Shock et al. reported a long transcript variant of human and mouse DNA methyltransferase 1 (*DNMT1*) that could translocate into mitochondria (mt*DNMT1*) and appear to be responsible for mtDNA cytosine methylation.^[^ ^22^ ^]^ Subsequently, the transcript isoform 3 of *DNMT1* was also observed to localize in mitochondria of human H1299 and HCT116 cells.^[^ ^25^ ^]^ In addition to DNMT1, other enzymes involved in DNA methylation, including DNMT3a,^[^ ^26^, ^27^, ^28^ ^]^ DNMT3b,^[^ ^23^, ^29^ ^]^ TET1 and TET2,^[^ ^30^ ^]^ have been detected in mitochondria in a tissue‐type dependent manner. Recently, based on the improved technology of whole genome bisulfite sequencing (WGBS) which is reported to effectively reduce the false‐positive detection,^[^ ^18^, ^20^, ^31^ ^]^ two surveys showed the genome‐wide mtDNA methylation profile of human tumor cell lines ^[^ ^29^ ^]^ and human brain ^[^ ^28^ ^]^ at single base resolution, providing strong support for the existence of mtDNA methylation. More importantly, some findings have indicated the potential association between mtDNA methylation and mitochondrial transcription,^[^ ^12^, ^25^, ^28^, ^29^, ^32^ ^]^ suggesting a novel avenue to modulate mtDNA‐encoded gene expression via epigenetic layer. Abnormal mtDNA methylation has been reported to be associated with several diseases and to frequently occur in the early stage of diseases and thus be used as a potential biomarker and diagnostic tool.^[^ ^12^, ^29^, ^33^ ^]^ In contrast, the biological significance and the detailed mechanisms involved are largely unexplored. Moreover, whether mtDNA methylation participates in IR and T2DM remains poorly understood.

In the present study, we unveiled a novel link between hypermethylation‐induced ND6 deficiency and IR in T2DM patients. We confirmed the specific hypermethylation of *ND6* in diabetic condition, contributing to suppressed *ND6* expression and mitochondrial dysfunction, which lead to systemic IR. Moreover, we observed that the *ND6* hypermethylation was attributed to increased DNMT1 mitochondrial localization, which was promoted by free fatty acid via activation of adenosine 5’‐monophosphate (AMP)‐activated protein kinase (AMPK). Using several complementary experimental strategies, we determined that inhibition of hepatic DNMT1 activity improved systemic insulin sensitivity and metabolic health in mice. We concluded that intervention of mitochondrial epigenetic regulatory network may be an effective therapeutic approach for insulin resistance associated metabolic disorders.

## 2 Results

### 2.1 *ND6* Hypermethylation in Human Peripheral Leukocytes is a Distinctive Biomarker for T2DM

**Figure 1:**
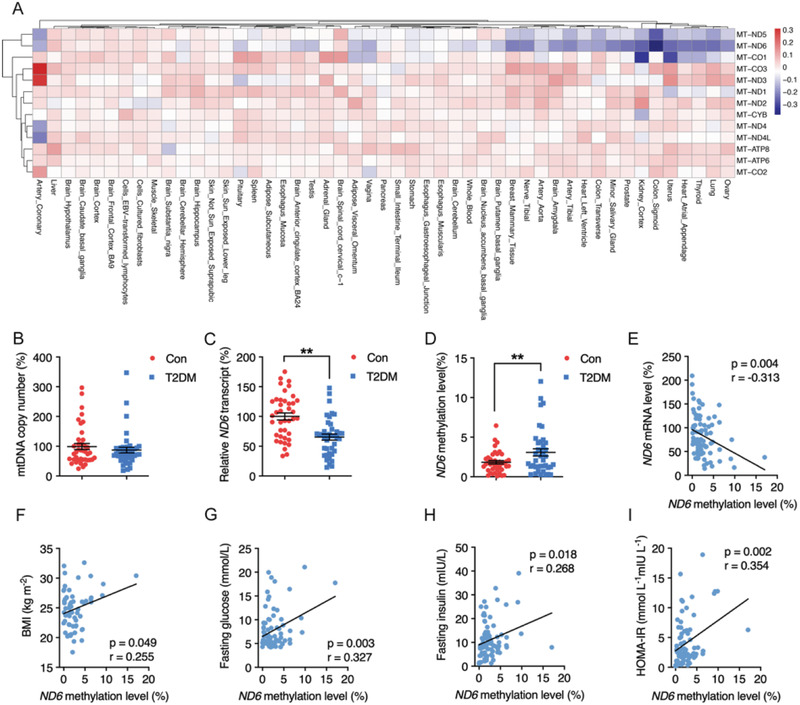
Positive correlation of *ND6* methylation with clinical insulin resistance status in human peripheral leukocytes. A) Heatmap of differential mRNA expression of 13 mitochondrial DNA (mtDNA) coding genes between obese subjects (body mass index (BMI) ≥ 30) and others (BMI < 30) across tissues, the log2 (fold change) value decreases with color changes from dark red to dark blue, *n* = 979. B–D) Relative mtDNA copy number, *ND6* mRNA level, and *ND6* methylation level in human peripheral leukocytes of non‐type 2 diabetes mellitus (non‐T2DM) and T2DM subjects, *n* = 39 for each group. E–I) Linear correlations between *ND6* mRNA and *ND6* methylation level (*n* = 78), BMI and *ND6* methylation level (*n* = 59), fasting glucose and *ND6* methylation level (*n* = 78), fasting insulin and *ND6* methylation level (*n* = 78), homeostasis model assessment‐insulin resistance (HOMA‐IR) index and *ND6* methylation level (*n* = 78) in human peripheral leukocytes of both non‐T2DM and T2DM subjects *n* = 78. Values are mean ± SEM **p < 0.01.

Obesity is considered as a central risk for T2DM.^[^ ^3^ ^]^ By analyzing the online data from the Genotype‐Tissue Expression (GTEx) project (release v8), we performed a heatmap for differential mRNA expression of 13 mtDNA‐encoded OXPHOS complex subunit genes in 49 tissues between obese subjects (BMI ≥ 30) and others (BMI < 30). Our analysis showed that among the 13 mtDNA encoded genes, *ND6* was the most significantly downregulated gene in majority of the tissues of obese subjects (**Figure** **1**A), suggesting *ND6* may serve as one of the most sensitive biomarkers in obesity. Based on the clues obtained from obese subjects, we therefore speculated that *ND6* may also play an important role in T2DM. Since no clinical features were indicated within the GTEx project, clinical peripheral leukocytes were collected from a cohort of donors with or without T2DM to further explore the involvement of *ND6* in the development of T2DM (Figure S1A–D, Supporting Information). Analysis showed no difference on mtDNA copy number between two groups (Figure 1B), while *ND6* mRNA presented a predominant decrease among 13 mtDNA encoded transcripts in diabetic group (Figure 1C and Figure S1E: Supporting Information) with nDNA encoded OXPHOS subunits unchanged (Figure S1F, Supporting Information), suggesting a potential unique role of *ND6* in metabolic regulation. Since mtDNA‐related transcription factors showed no changes between groups (Figure S1F, Supporting Information), and mtDNA methylation was recently suggested to mediate mitochondrial transcription,^[^ ^12^, ^25^, ^28^, ^29^, ^32^ ^]^ we thereby analyzed the methylation level of *ND6* in the peripheral leukocytes through methylation‐specific PCR. And data showed that *ND6* methylation was significantly higher in T2DM patients compared to the control (Figure 1D). Notably, *ND6* methylation was inversely correlated with *ND6* mRNA level while positively correlated with BMI, fasting glucose, fasting insulin, and homeostasis model assessment‐insulin resistance (HOMA‐IR) index (Figure 1E–I). Further group specific regression analysis revealed that the dominant contribution was primarily attributed to T2DM group except for BMI correlation (Figure S1H–Q, Supporting Information). In addition, D‐loop methylation was also found significantly increased in T2DM group (Figure S1G, Supporting Information), while the linear correlations between the methylation level of D‐loop and the above clinical parameters were not significant except for the BMI (Figure S1R–U, Supporting Information). Taken together, these data suggest that the increased *ND6* methylation in peripheral leukocytes might serve as a distinctive predictor for T2DM.

### 2.2 *ND6* is a Primary Target of mtDNA Methylation in the Liver of Insulin Resistant Mice

**Figure 2:**
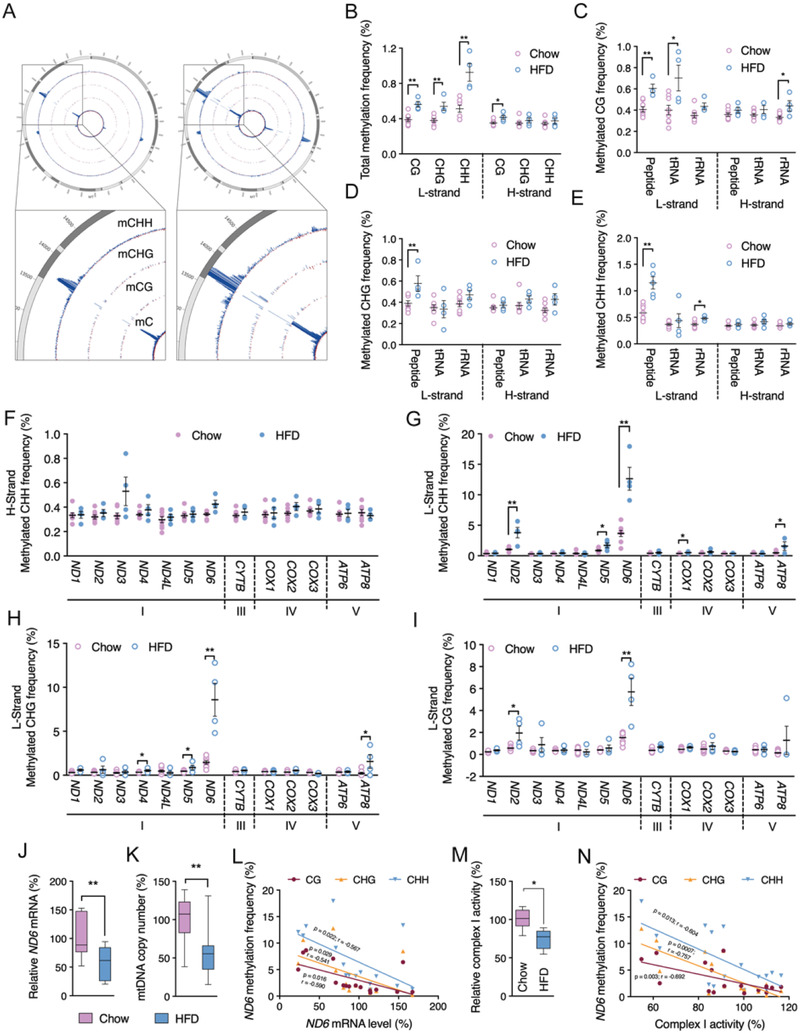
*ND6* is a primary target of mtDNA methylation in the liver of high fat diet (HFD) induced insulin resistant mice. A) Genome‐wide scale view of methylated C, CG, CHG, and CHH frequencies on both L and H strands of liver mtDNA. The blue lines indicate methylated C, CG, CHG, and CHH frequencies on L‐strand, and the red lines indicate methylation on H‐strand. B) Total methylated CG, CHG, and CHH frequencies. C–E) Methylated CG, CHG, and CHH frequencies of peptides, tRNAs and rRNAs on liver mtDNA. F–I) Methylated CHH frequencies on H‐strand, methylated CHH, CHG, and CG frequencies on L‐strand of 13 oxidative phosphorylation (OXPHOS) complex subunits. J) Relative liver *ND6* mRNA level. K) Relative liver mtDNA copy number. L) Linear correlation between liver *ND6* mRNA level and *ND6* L‐strand methylated CG, CHG, and CHH frequencies. M) Relative liver complex I activity. N) Linear correlation between liver complex I activity and *ND6* L‐strand methylated CG, CHG, and CHH frequencies. Values are mean ± SEM. In B–K and M, *n* = 4 for each group; in L and N, *n* = 16. *p < 0.05, **p < 0.01.

Given the central role of liver during the onset of IR, we further explore the involvement of hepatic *ND6* methylation in the progression of metabolic disorder. HFD‐induced insulin resistant mice and db/db mice were employed as animal models of liver IR (Figure S2A–D, Supporting Information). mtDNA was isolated from mouse liver (Figure S2E, Supporting Information) for genome‐wide bisulfite sequencing at single base resolution. Consistent with previous reports,^[^ ^23^, ^24^ ^]^ presence of mtDNA methylation was confirmed in mouse liver with all three sequence contexts including methylated mtDNA CpG (CG) and non‐CpG (CHG and CHH) on both L and H strands (**Figure** **2**A). Interestingly, the methylation showed unique features, including overall low methylation frequencies on both strands of mtDNA for most sites (<1%), asymmetric methylation patterns on both strands, and higher frequencies on L‐strand with particular significance in the CHH sequence in both HFD and db/db mice model (Figure 2A,B and Figure S2F, Supporting Information). Comparing to normal control, HFD group presented consistently higher methylation on peptides encoding region of L‐strand at all three sequence contexts (Figure 2C–E), while increased methylation on tRNA and rRNA was only spotted at CG and CHH contexts of L‐strand, respectively. Comparing to C57 control, db/db mice group presented higher methylation peptides region of L‐strand at only CG and CHG contexts, methylation of tRNA and rRNA was not affected (Figure S2G–I, Supporting Information).

**Figure 3:**
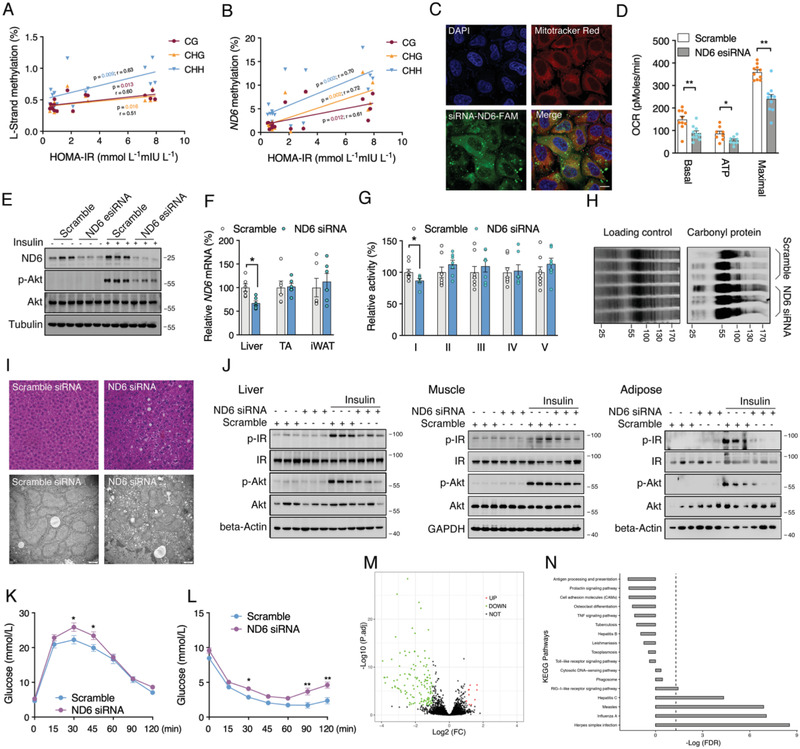
*ND6* knockdown results in mitochondrial dysfunction and insulin resistance. A,B) Linear correlations between HOMA‐IR index and liver mtDNA L‐strand and *ND6* L‐strand methylation (*n* = 16). C) Fluorescence co‐localization *ND6* siRNA‐FAM and mitochondria in 3T3‐L1 cells. D) Mitochondrial oxygen consumption rate (OCR) of HepG2 cells transfected with *ND6* esiRNA for 48 h (*n* = 9). E) Protein levels of ND6, p‐Akt, and Akt in *ND6* knockdown HepG2 cells followed by insulin challenge for 10 min (*n* = 3). F) Relative *ND6* mRNA levels in the liver, tibialis anterior muscle (TA) and inguinal white adipose tissue (iWAT) (*n* = 6); G) Relative liver mitochondrial complex activities (*n* = 6); H) Liver mitochondrial carbonyl protein level (*n* = 3); I) Liver hematoxylin‐eosin (HE) staining and electron microscopy images in *ND6* siRNA‐treated mice. J) Protein levels of p‐IR, IR, p‐Akt, and Akt in the liver, TA, and iWAT of *ND6* siRNA‐treated mice with or without insulin challenge (*n* = 3). K,L) Oral glucose tolerance test (OGTT) and insulin tolerance test (ITT) analysis in *ND6* siRNA‐treated mice (*n* = 6). M,N) RNA‐sequencing with the volcano plots and the Kyoto Encyclopedia of Genes and Genomes (KEGG) pathways analysis of mice liver (*n* = 4). Values are mean ± SEM, *p < 0.05, **p < 0.01.

To further evaluate the differential methylation patterns of 13 mtDNA encoding genes for peptide, average CHH methylation frequencies in control and HFD groups were analyzed. Consistent with the total methylation level, no significant alterations of specific genes showed on the H‐strand (Figure 2F), while the L‐strand CHH frequency presented significant increase on *ND2*, *ND5*, *ND6*, *COX1*, and *ATP8* methylation (Figure 2G). Meanwhile, CHG and CG methylation context also showed increase on specific genes of L‐strand (Figure 2H,I). Consistently, db/db mice showed no significant alterations of specific genes on the H‐strand comparing to C57 control (Figure S2J, Supporting Information). Increased *ND1*, *ND6*, *CYTB*, *COX1* at CG context, *ND2*, *ND6*, *COX1* at CHG context, and *ND6* at CHH context was observed on the L‐strand of db/db mice (Figure S2K–M, Supporting Information). Despite different types of methylation on specific genes, the *ND6* methylation frequency showed a dramatic and consistent increase on the L‐strand under HFD or genetic induced insulin resistant condition, representing the highest methylation level among all the regions of the mtDNA. We thereby assume that *ND6* is the primary target of mtDNA methylation in insulin resistant mice. *ND6* mRNA was found significantly decreased in the liver of insulin resistant mice in addition to decreased mtDNA copy number (Figure 2J,K and Figure S2N,O: Supporting Information). Linear regression calculated a consistent inverse correlation between the *ND6* mRNA level and the frequencies of all three methylation sequence contexts including CG, CHG and CHH (Figure 2L), suggesting that *ND6* methylation inversely regulate the transcription activity. As expected, the downregulated *ND6* expression resulted in lowered complex I activity in insulin resistant mouse liver (Figure 2M and Figure S2P: Supporting Information), which was confirmed by significant inverse correlation between complex I activity and *ND6* methylation frequencies (Figure 2N). In contrast, none significant correlation was observed between complex I activity and D‐loop methylation frequencies with all three sequence contexts (CG, CHG, and CHH) (Figure S3A–C, Supporting Information). These data suggest a critical role of *ND6* in maintaining the complex I activity. Meanwhile, a strong positive correlation was found between the HOMA‐IR index and mtDNA overall CG, CHG, and CHH methylation on the L‐strand expect for D‐Loop region (**Figure** **3**A and Figure S3D,E: Supporting Information), and significant correlation of HOMA‐IR was also observed for the *ND6* L‐strand methylation, highlighting the vital role of *ND6* methylation during the progression of IR (Figure 3B).

### 2.3 *ND6* Knockdown Results in Mitochondrial Dysfunction and IR

In order to confirm the regulatory role of *ND6* in IR, both in vitro and in vivo studies on *ND6* knockdown were carried out. Given the support from recent studies showing successful import of exogenous RNAs into mitochondria,^[^ ^34^, ^35^ ^]^ a pool of *ND6* siRNAs was under screening for knockdown efficiency. Confocal microscopy indicated a significant population of *ND6* siRNA in the mitochondria, suggesting a potential effective strategy for knockdown of *ND6* (Figure 3C). In vitro transfection of *ND6* esiRNA significantly knockdown *ND6* expression and impaired mitochondrial oxygen consumption rate (OCR) function as well as insulin sensitivity in HepG2 cells (Figure 3D,E). Consistent results were also observed in murine 3T3‐L1 and HT22 cells transfected with *ND6* siRNAs (Figure S4A,B: Supporting Information), with robustly increased mitochondrial superoxide level (Figure S4C, Supporting Information). *ND6* siRNA was processed with agomir‐modification for mouse delivery via tail vein injection. Since skeletal muscle and adipose tissue are another two organs that closely involved in the development of insulin resistance in addition to liver, we thereby collected tissues from liver, tibialis anterior muscle (TA) and inguinal white adipose tissue (iWAT) for analysis of *ND6* mRNA to validate the knockdown specificity. As expected, down‐regulation of *ND6* mRNA was observed in liver, while not in TA and iWAT (Figure 3F), indicating the majority accumulation of the *ND6* siRNA in liver, which was consistent with previous study.^[^ ^36^ ^]^ Further analysis of all 13 mtDNA encoded transcripts and part of nDNA encoded complex subunits confirmed specific dowregulated expression of *ND6* (Figure S4D,E: Supporting Information). 8‐day *ND6* siRNA treatment did not affect body and tissue weight (Figure S4F, Supporting Information), but significantly lowered complex I activity in liver without affecting other complexes (Figure 3G). In line with the above results, *ND6* knockdown largely elevated mitochondrial carbonyl protein level in the liver (Figure 3H), consistent with previous reports linking dysregulated complex I with mitochondrial oxidative stress.^[^ ^37^, ^38^ ^]^ In addition, transmission electron microscopy displayed more fragmented and disoriented mitochondria in the liver of *ND6* siRNA‐treated mice accompanied with slightly increased lipid deposition (Figure 3I), which was consistent with elevated liver triglycerides (TG) level (Figure S4G, Supporting Information). However, treatment with *ND6* siRNA had no effects on total cholesterol (T‐CHO) level in liver as well as serum lipids (Figure S4H–K, Supporting Information). Impaired mitochondrial function is reported to be closely associated IR,^[^ ^2^, ^3^, ^4^, ^5^ ^]^ which was confirmed in the liver of *ND6* siRNA treated mice under insulin challenge (Figure 3J). Interestingly, the suppressed insulin sensitivity was also observed in the mouse TA and iWAT (Figure 3J), suggesting a central role of liver in regulating systemic insulin sensitivity. Further oral glucose tolerance test (OGTT) and insulin tolerance test (ITT) consolidated the decrease of insulin sensitivity induced by *ND6* knockdown (Figure 3K,L). To ensure the on‐target effects of *ND6* siRNA, RNA sequencing of liver samples identified only a total of 336 differential genes, which showed strong association with siRNA transfection‐related pathways with no direct changes in insulin related regulatory network (Figure 3M,N). Taken together, the above data further elucidated the substantial role of hepatic *ND6* in modulating mitochondrial function and systemic IR.

### 2.4 Mitochondrial DNMT1 Promoted‐Hepatic *ND6* Hypermethylation Triggers IR

**Figure 4:**
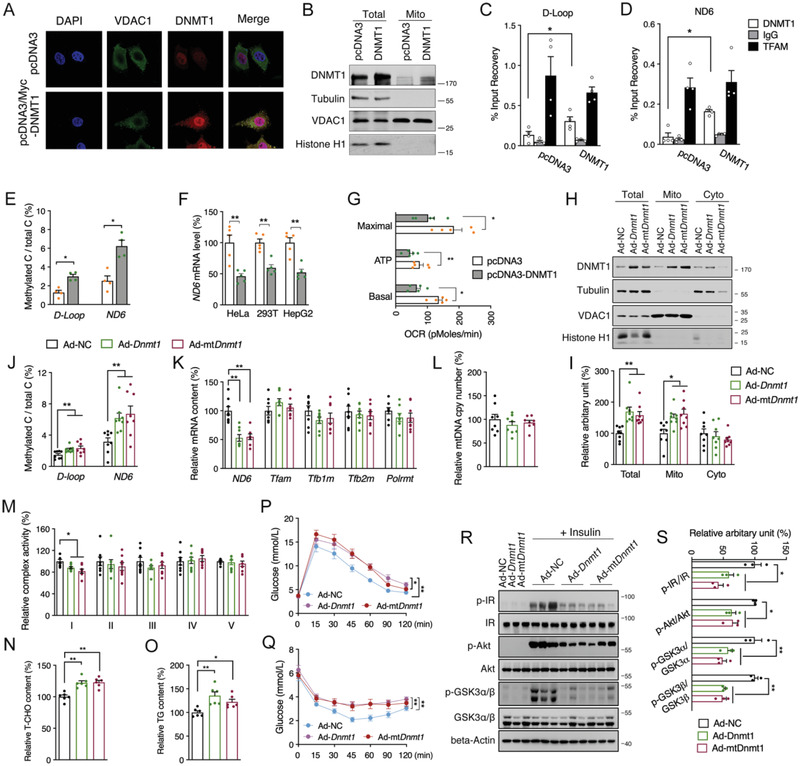
Mitochondrial DNMT1‐promoted hepatic *ND6* hypermethylation triggers insulin resistance. A) Co‐localization DNMT1 and mitochondria in HepG2 cells. B) Western blot analysis of DNMT1 protein level in mitochondrial fraction of HepG2 cells. C,D) mtDNA immunoprecipitation (mtDIP) assay performed for DNMT1 binding to mtDNA D‐loop and *ND6* in transfected HepG2 cells (*n* = 4). E) Methylated D‐loop and *ND6* levels of transfected HepG2 cells (*n* = 4). F) Relative *ND6* mRNA levels in HeLa, 293T and HepG2 cells transfected with pcDNA3 and pcDNA3‐*DNMT1* for 48 h (*n* = 4). G) Mitochondrial OCR of transfected HepG2 cells (*n* = 5). H,I) Liver DNMT1 protein level in total, mitochondrial and cytosolic fractions of mice infected with pAd/CMV/V5‐*Dnmt1*(Ad‐*Dnmt1*), pAd/CMV/V5‐mt*Dnmt1*(Ad‐mt*Dnmt1*) or pAd/CMV/V5‐NC (Ad‐NC) adenovirus via the tail vein injection (*n* = 8). J) Methylation level of mtDNA D‐loop and *ND6*; K) Relative mRNA levels of *ND6*, *Tfam*, *Tfb1m*, *Tfb2m*, and *Polrmt*; L) Relative mtDNA copy number; M) Relative complex activities; N) Relative total cholesterol (T‐CHO); O) Relative triglycerides (TG) in the liver of adenovirus infected mice (*n* = 8). P,Q) OGTT and ITT analysis in mice (*n* = 8). R,S) Hepatic protein levels of p‐IR, IR, p‐Akt, Akt, p‐GSK3*α*/*β* and GSK3*α*/*β* in adenovirus infected mice treated with or without insulin (*n* = 3). Values are mean ± SEM. *p < 0.05, **p < 0.01.

Next, we aimed to explore the upstream network for modulating *ND6* methylation. Subcellular fraction analysis showed significantly increased mitochondrial localization of liver DNMT1 in both HFD and db/db mice, while DNMT3a was unchanged and DNMT3b was not detectable in mitochondria (Figure S5, Supporting Information), indicating a potential role of DNMT1 in regulating mtDNA methylation. Immunofluorescence imaging indicated that overexpression of wild‐type *DNMT1* promoted both mitochondrial and nuclear localization in HepG2 cells (**Figure** **4**A), which was further confirmed by western blot of mitochondrial fractions (Figure 4B). mtDNA immunoprecipitation (mtDIP) also exhibited significantly increased binding of DNMT1 to mtDNA D‐loop and *ND6* regions after *DNMT1* overexpression (Figure 4C,D). Consistently, methylation levels of *ND6* as well as D‐loop were highly elevated (Figure 4E), while the *ND6* mRNA was significantly decreased in HepG2 and other cell lines including HeLa and 293T (Figure 4F), indicating a universal regulation of DNMT1 on *ND6* expression. Consistent with *ND6* siRNA knockdown, *DNMT1* overexpression in HepG2 cells sufficiently impaired mitochondrial function (Figure 4G).

To explore the involvement of DNMT1 mediated *ND6* expression and insulin signaling in vivo, wild‐type *Dnmt1* (*Dnmt1*) and variant *Dnmt1* carrying the mitochondrial targeting sequence (mt*Dnmt1*) was constructed into adenovirus vectors. Meanwhile, a control adenovirus vector with *GFP* tag was employed to determine the distribution of adenovirus in mice via tail vein injection, tissue analysis showed predominant expression of *GFP* in liver (Figure S6A, Supporting Information). After injection of *Dnmt1* or mt*Dnmt1* adenovirus for 12 days, we observed significantly up‐regulated DNMT1 in both mRNA and protein levels in the liver (Figure S6B–D, Supporting Information), and neither the body weight nor the liver or other organ weights showed apparent alterations (Figure S6E, Supporting Information). As expected, increased translocation of DNMT1 into mitochondria was confirmed in the liver infected with both *Dnmt1* and mt*Dnmt1* adenovirus, suggesting that the localization of DNMT1 to mitochondria may not entirely depend on the mitochondrial targeting sequence (Figure 4H,I). Consequently, hepatic *ND6* methylation level was increased and *ND6* expression was decreased without affecting mtDNA copy number and other nDNA encoded subunits (Figure 4J–L and Figure S6F: Supporting Information), followed by specific suppression of Complex I activity (Figure 4M) and increases of T‐CHO and TG deposition in liver (Figure 4N,O), while serum lipids were not altered (Figure S6G–J, Supporting Information). Finally, consistent with direct hepatic *ND6* knockdown, *Dnmt1* overexpression sufficiently impaired mouse glucose tolerance and insulin sensitivity (Figure 4P,Q), which was further consolidated with suppressed hepatic Akt signaling (Figure 4R,S). Collectively, both in vitro and in vivo data imply a direct regulation of DNMT1 on *ND6* methylation and transcription, which plays vital role in mitochondrial dysfunction associated hepatic IR.

### 2.5 Free Fatty Acid Promotes DNMT1 Mitochondrial Localization via Activation of AMPK

**Figure 5:**
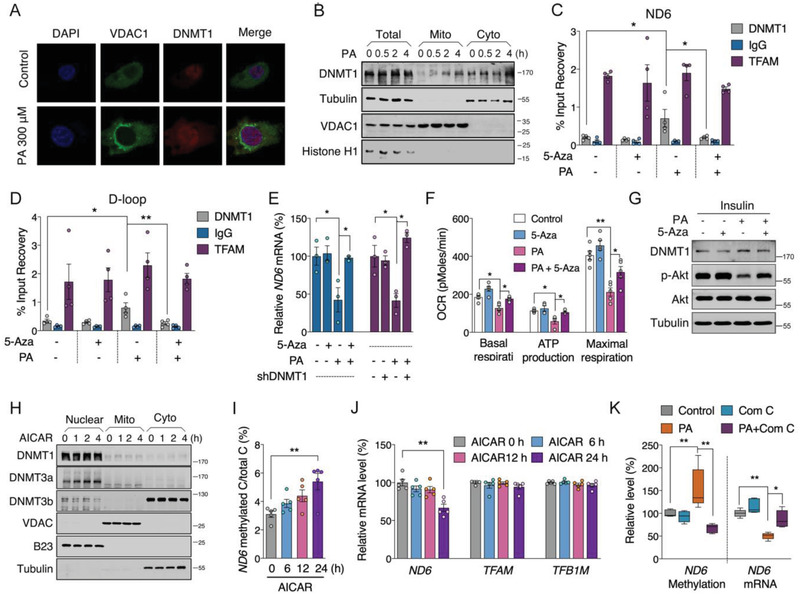
Palmitic acid (PA) induces hepatic insulin resistance via DNMT1 associated *ND6* hypermethylation. A) Localization of DNMT1 in PA treated HepG2 cells. B) Western blot assay of DNMT1 protein level in mitochondrial and cytosolic fractions of PA treated HepG2 cells. C,D) mtDIP assay for DNMT1 binding to mtDNA *ND6* and D‐loop in PA treated HepG2 cells with or without 2 × 10^−6^ m 5‐Aza‐2’‐deoxycytidine (5‐Aza) (*n* = 4). E) Relative *ND6* mRNA level in PA treated HepG2 cells treated with or without inhibition of DNMT1 through 5‐Aza or sh*DNMT1* transfection (*n* = 3). F) Mitochondrial OCR analysis after PA and 5‐Aza treatment (*n* = 5). G) Western blot analysis of DNMT1, p‐Akt, and Akt levels in PA treated HepG2 cells with or without 5‐Aza followed by insulin stimulation for 10 min. H) Western blot analysis of DNMT1, DNMT3a, DNMT3b subcellular localization in HepG2 cells treated with 1 × 10^−3^ m 5‐aminoimidazole‐4‐carboxamide 1‐*β*‐D‐ribofuranoside (AICAR). I,J) Methylation level of *ND6* and mRNA levels of *ND6*, *TFAM* and *TFB1M* in AICAR treated cells (*n* = 5). K) *ND6* methylation and mRNA levels in PA treated cells with or without 5 × 10^−6^ m Compound C (Com C) for 24 h (*n* = 5). Values are mean ± SEM. *p < 0.05, **p < 0.01.

As enriched free fatty acid was the major cause for HFD‐induced fatty liver and IR,^[^ ^39^ ^]^ we speculated that DNMT1 was the effective target of free fatty acid in the process of liver pathology. Palmitic acid (PA) was found to time‐ and dose‐dependently decrease *ND6* expression in HepG2 cells (Figure S7A,B: Supporting Information). Consistent with observations in insulin resistant mice, enhanced mitochondrial translocation of DNMT1 was observed after short time PA treatment in both HepG2 cells and mouse primary hepatocytes (**Figure** **5**A,B and Figure S7C: Supporting Information), suggesting DNMT1 mediated *ND6* hypermethylation being an early event during onset of mitochondrial dysfunction and IR. Notably, employment of the DNMT1 inhibitor 5‐Aza‐2’‐deoxycytidine (5‐Aza) significantly decreased the elevated binding of DNMT1 to mtDNA *ND6* and D‐loop region induced by PA (Figure 5C,D), lowered *ND6* and D‐loop methylation levels (Figure S7D, Supporting Information), followed by improved *ND6* expression (Figure 5E), mitochondrial OCR capability (Figure 5F) and insulin sensitivity (Figure 5G). Consistent recovered *ND6* transcription was also observed in *DNMT1* knockdown HepG2 cells (Figure 5E). These data suggest that PA‐induced mitochondrial dysfunction and IR through promoting DNMT1 activity in mitochondria.

The AMPK pathway and mitogen‐activated protein kinase (MAPK) pathway are reported to be closed linked to PA‐induced IR.^[^ ^40^ ^]^ We thus reasoned that certain kinases were involved in PA‐promoted DNMT1 translocation within a short time. By treatment of HepG2 cells with PA in a time‐dependent manner, we found that among all the phosphokinases, only the phosphorylations of AMPK and Akt were efficiently elevated within 2 h (Figure S7E, Supporting Information), consist with previous report revealing that AMPK could regulate DNMT1 activity.^[^ ^41^ ^]^ To specifically confirm the role of AMPK in PA‐regulated DNMT1 activity in mitochondria, AMPK activator 5‐aminoimidazole‐4‐carboxamide 1‐*β*‐D‐ribofuranoside (AICAR) was used to treat HepG2 cells and was found to efficiently promote DNMT1 mitochondrial localization within 4 h (Figure 5H), followed by increased *ND6* methylation and decreased *ND6* mRNA level within 24 h without affecting mtDNA copy number and related transcription factors (Figure 5I,J and Figure S7F : Supporting Information). Interestingly, AICAR induced *ND6* mRNA decrease was restored after 48 h‐prolonged treatment, indicating that AMPK dynamically regulated DNMT1 mitochondrial activity (Figure S7G, Supporting Information), while the physiological role of short‐term AICAR effect on *ND6* expression remains further investigation. However, we found no obvious changes of mtDNA copy number and *ND6* mRNA level after treatment of Akt activator SC79 in HepG2 cells, indicating Akt may not be the major upstream regulator of DNMT1‐mediated *ND6* hypermethylation (Figure S7H,I: Supporting Information). In addition, inhibiting AMPK with compound C sufficiently reversed the *ND6* methylation and mRNA expression in PA treated cells (Figure 5K), while inhibiting Akt with LY294002 showed no changes (Figure S7J, Supporting Information), further consolidating the specificity of AMPK on PA‐modulated DNMT1 activity in mitochondria.

### 2.6 Suppression of DNMT1 Restores *ND6* Expression and Improves Insulin Sensitivity in Insulin Resistant Mice

**Figure 6:**
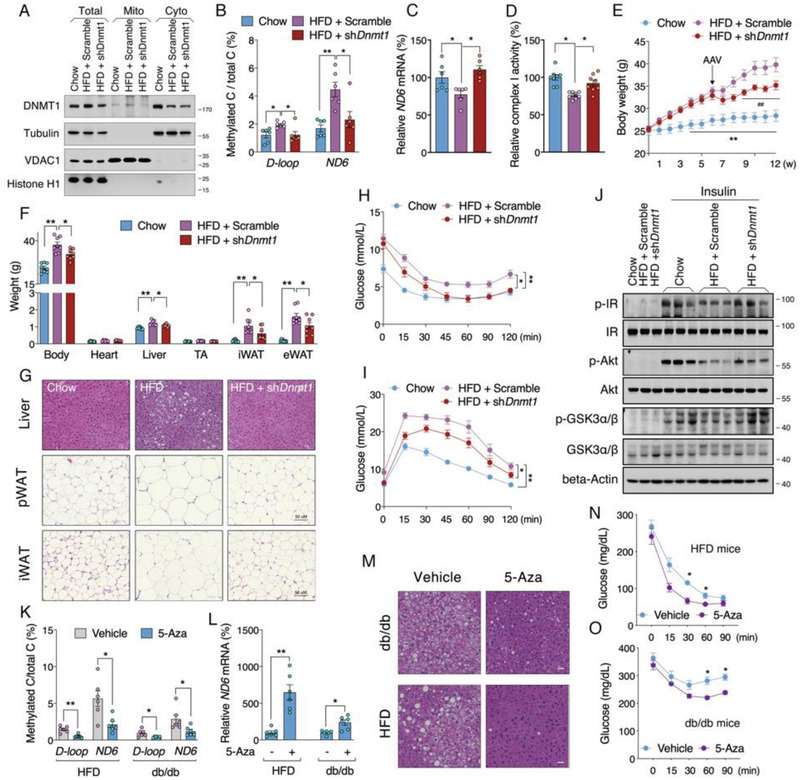
Suppression of DNMT1 restores *ND6* expression and improves insulin sensitivity in insulin resistant mice. A) Liver DNMT1 protein subcellular level in mice fed on normal diet or HFD infected with adeno‐associated viruses (Adeno‐associated virus serotype 8 (AAV8)‐*Dnmt1* shRNA) for *Dnmt1* knockdown. B) Methylated levels of D‐loop and *ND6* (*n* = 6); C) Relative *ND6* mRNA level (*n* = 6); D) Relative mitochondrial complex I activity (*n* = 8) in the liver of AAV treated mice fed on HFD. E) Body weight curve of AAV8 infected mice fed on HFD (*n* = 8). F) Body and tissue weights of mice after diets intervention for 12 weeks and AAV8 infection for 6 weeks (*n* = 8). G) HE staining of liver, perirenal white adipose tissue (pWAT) and inguinal white adipose tissue (iWAT) tissues. H,I) ITT and OGTT analysis in mice after the diet and AAV8 intervention (*n* = 8). J) Protein level of Akt signals in the liver of diets and AAV treated mice following insulin challenge for 15 min (*n* = 3). K,L) D‐loop and *ND6* methylation levels and relative *ND6* mRNA level in HFD and db/db mice with or without 5‐Aza administration (*n* = 6). M) HE staining of liver tissues. N,O) ITT analysis of HFD and db/db mice with or without 5‐Aza administration (*n* = 6). Values are mean ± SEM. *p < 0.05, **p < 0.01.

To explore the benefits of targeting hepatic DNMT1 in vivo, Adeno‐associated virus serotype 8 (AAV8) was selected to package the mouse *Dnmt1* shRNA for liver targeting. We found that the shRNA sufficiently downregulated liver DNMT1 protein and its mitochondrial localization (**Figure** **6**A). The increased methylation of D‐loop and *ND6* under HFD was dramatically restored to normal (Figure 6B). Consistent improvement was also observed on *ND6* mRNA (Figure 6C), without significant changes on mtDNA‐related transcription factors and nDNA‐encoded mitochondrial OXPHOS subunits (Figure S8A,B: Supporting Information). In addition, HFD decreased both complex I and complex II activities, while knockdown of *Dnmt1* specifically improved complex I activity (Figure 6D and Figure S8C : Supporting Information), suggesting a unique epigenetic regulation of complex I activity under HFD challenge. Notably, liver specific knockdown of *Dnmt1* could sufficiently lower the increase in body weight, as well as hepatic lipid deposition and adipocytes hypertrophy induced by HFD (Figure 6E–G), which was further supported by chemical analysis of lipids in liver, aminotransferase activities and lipids in serum (Figure S8D–K, Supporting Information). Moreover, HFD impaired insulin sensitivity and glucose tolerance were also improved by hepatic knockdown of *Dnmt1* (Figure 6H,I and Figure S8L: Supporting Information). Further analysis of insulin signal pathway exhibited recovered p‐IR, p‐Akt and p‐GSK3*α*/*β* levels upon insulin stimulation in the liver of *Dnmt1* shRNA‐treated mice (Figure 6J). Interestingly, hepatic *Dnmt1* knockdown failed to ameliorate HFD‐induced fasting insulin elevation, indicating a primary involvement of insulin sensitivity regulation by DNMT1 (Figure S8M, Supporting Information). Collectively, these data suggest an effective and beneficial strategy by targeting hepatic DNMT1 for systemic improvement of metabolic disorders. Since 5‐Aza was previously reported to inhibit DNA methylation by selective degradation of DNMT1,^[^ ^42^ ^]^ we also adopted 5‐Aza treatment in HFD and db/db insulin resistant animals. Data showed that 5‐Aza could also robustly increase *ND6* transcription by lowering methylation level (Figure 6K,L). Hepatic lipid deposition and insulin sensitivity were also effectively improved by 5‐Aza intervention (Figure 6M–O). Taken together, our data proposed a pivotal role of *ND6* methylation during onset of IR, therefore, targeting its epigenetic network could be feasible for treatment of IR and type‐2 diabetes.

## 3 Discussion

Despite awareness of nuclear epigenetic factors in modulating the risk of IR,^[^ ^13^ ^]^ the mitochondrial epigenetic regulation and involved molecular mechanisms remain largely obscure and overlooked due to the challenge of technology. In the present study, we show that L‐strand specific hypermethylation of *ND6* is a distinctive epigenetic event in both T2DM patients and insulin resistant mice, which was closely correlated with decreased *ND6* transcription and impaired OXPHOS function. Thus, elevated *ND6* methylation may be a promising biomarker with predictive and diagnostic value for T2DM. The present study also illustrates the genome‐wide patterns of frequencies of both methylated mtDNA CpG (CG) and non‐CpG (CHG and CHH) at single‐base resolution in insulin resistant model for the first time and reveals L‐strand‐biased visible alterations of mtDNA methylation levels and distributions. Furthermore, we demonstrate that the hypermethylation of *ND6* is attributed to increased DNMT1 mitochondrial localization, which can be promoted by elevated circulating free fatty acid via AMPK activation, suggesting that AMPK/DNMT1 axis may be a potential therapeutic target for the prevention and treatment of IR and T2DM.

As one of the most important aspects of mitochondrial epigenetics, mtDNA methylation has been debated since 1970s.^[^ ^14^, ^15^, ^16^ ^]^ Part of previous studies claimed that the methylation level of mtDNA may be overestimated due to bisulfite conversion inefficiency triggered by the secondary structure of mtDNA.^[^ ^18^, ^20^, ^31^ ^]^ While, recent technology improvements raise the possibility to reliably detect the mtDNA methylation.^[^ ^28^, ^29^ ^]^ Virtually, there are two key points for successful mtDNA methylation detection: the separation of mtDNA from nuclear DNA and the elimination of mtDNA secondary structures before bisulfate conversion. To increase the enrichment of mtDNA, we adopted Exonuclease V digestion followed by phenol‐chloroform extraction and Ampure beads to remove nuclear DNA and proteins. The purity of mtDNA was confirmed by real‐time PCR analysis using specific mitochondrial and nuclear primers. In addition, to avoid the secondary structures, all the mtDNA samples were fragmented prior to bisulfite conversion to perform further methylation‐specific PCR or sequencing. The above two crucial steps consolidated the reliability of mtDNA methylation detection in our study.

mtDNA‐encoded 13 proteins are essential to maintain OXPHOS function.^[^ ^6^ ^]^ It has been reported that a deficiency of mitochondrial OXPHOS plays a vital role in the development of IR,^[^ ^4^, ^5^ ^]^ whereas how mtDNA‐encoded genes participate in metabolic disorders such as obesity and T2DM remains largely unclear. Recent evidence revealed a novel possibility of mtDNA methylation in the regulation of mitochondrial transcription,^[^ ^12^, ^25^, ^28^, ^29^, ^32^ ^]^ which prompted us to explore the regulatory role of mtDNA methylation in IR and T2DM. Though obesity is not equal to T2DM, but is well accepted as one of the key risk factors for developing T2DM. The present study was initiated to analyze mtDNA encoded subunits expression pattern with online database GTEx project which only characterize the attendants with BMI. From the data showed, we observed a decrease of *ND6* expression in multiple tissues of obese subjects. Since no clinical features were indicated within the GTEx project and hepatic insulin resistance is a long‐term and chronic progress following obesity, we further collected peripheral leukocytes from healthy and T2DM subjects and confirmed the dramatic decrease of *ND6* expression with associated hypermethylation in T2DM subjects. Above two studies with human subjects gave us the clue that *ND6* expression might be closely involved in the development of insulin resistance, which led us to employ progressive animal (HFD‐induced and non‐diet dependent insulin resistance mice) and cellular models to explore the details. We found significantly and specifically decreased *ND6* transcription under diabetic condition which exhibited close negative correlation with the elevated *ND6* methylation level. It is noteworthy that the *ND6* methylation level was also positively correlated with clinical features driving a close link between *ND6* hypermethylation and IR. However, the D‐loop methylation level was only correlated with BMI, which was in line with the recent study.^[^ ^43^ ^]^ Although the lack of human hepatic *ND6* expression pattern in T2DM population presents a limitation of the study, we have substantial evidence to demonstrate that instead of obesity itself, a direct link lies between elevated free fatty acid and hepatic insulin resistance, which we suggest playing a vital role during the onset of high calorie high fat associated insulin resistance.

The genome‐wide profile of mammalian mtDNA methylation has not been reported in metabolic disorders. Thus, we mapped the liver mtDNA methylome in C57BL/6J control, HFD and db/db mice, and observed elevated mtDNA methylation frequencies in insulin resistant liver especially in the region encoded by the *ND6* gene (13552‐14070). Furthermore, the methylation changes were more apparent in HFD mice than in db/db mice, which indicated a more important role of diet‐mediated epigenetic alteration during disease progression. Notably, both the frequencies and alterations of methylation occurred predominantly on the L‐strand compared with the H‐strand particularly in CHH sequence, and these L‐strand‐biased non‐CpG methylation features of mtDNA were consistent with recent studies,^[^ ^28^, ^29^ ^]^ which may partially due to the more abundant C sites on the L‐strand and in non‐CpG context. Compared with CpG methylation, the physiological significance of non‐CpG methylation is largely unknown.^[^ ^44^ ^]^ Here, we showed that both *ND6* transcription and mitochondrial complex I activity exhibited significant associations with *ND6* CG, CHG and CHH methylation frequencies on the L‐strand, supporting the involvement of non‐CpG methylation in physiological regulation. Since ND6 is encoded by the L‐strand, we thereby assume that the increased methylation level of the L‐strand inhibits *ND6* transcription. More efforts shall be dedicated to exploring the detailed mechanism of non‐CG methylation in regulation of mtDNA‐encoded gene transcription. In addition, recent studies have reported that the methylation of CG, CHG, and CHH patterns could also occur in the regions of mtDNA, tRNA, and rRNA, especially on the L‐strand,^[^ ^28^, ^29^ ^]^ while their functional relevance remains largely unknown. In the present study, we also observed increased methylation on tRNA and rRNA at CG and CHH contexts of L‐strand in the liver of HFD group. Further investigations are required to explore the detailed regulatory role of mtDNA tRNA and rRNA hypermethylation in the occurrence of insulin resistance.

DNMT1 was initially discovered as a nuclear protein regulating nuclear DNA methylation, but the detailed mechanism accounting for DNMT1 mitochondrial translocation remains elusive. Shock et al. ^[^ ^22^ ^]^ reported a transcript variant of human and mouse *DNMT1* that containing an additional N‐terminal sequence to facilitate DNMT1 translocate into mitochondria. Later, the shortest isoform of human DNMT1 (isoform 3) was also observed to be able to import into mitochondria.^[^ ^25^ ^]^ In order to assess the probability of DNMT1 to target mitochondria, all the human and mouse DNMT1 isoforms were predicted by MitoProt II (Table S1, Supporting Information). Interestingly, despite the mtDNMT1 isoforms reported by Shock et al. ^[^ ^22^ ^]^ had the highest probability of import to mitochondria (85.54% for human mtDNMT1 and 91.70% for mouse mtDNMT1; 92.26% for human TFAM as the positive control), all the other human or mouse wild‐type DNMT1 isoforms published in NCBI also had probabilities from 36.13% to 72.45% to translocate into mitochondria with a predicted 10 or 20 amino acids of the mitochondrial leader peptide in their original N‐terminal. In line with the predicted results, we found that overexpression of human wild‐type *DNMT1* (NM_0 01379.4) could still increase its protein level in mitochondria of HepG2 cells, which confirmed by the elevated binding of DNMT1 to mtDNA D‐loop and *ND6* regions and the increased methylation levels of D‐loop and *ND6*. More importantly, hepatic overexpression of wild‐type *Dnmt1* (NM_0 011 99431.1) or mt*Dnmt1* (NM_0 011 99431.1 tagged with an additional mitochondrial targeting sequence) respectively via tail vein injection could cause similar phenotypes including elevated mitochondrial DNMT1 protein level and *ND6* methylation level, reduced *ND6* mRNA content and Complex I activity, and impaired insulin sensitivity, suggesting that the localization of DNMT1 to mitochondria may not depend only on the transcript variant carrying the mitochondrial targeting sequence.

Though DNMT1 was confirmed with its mitochondrial localization, the upstream mechanism of promoting the import of DNMT1 to mitochondria remains largely unclear. As a common energy sensor, AMPK has been demonstrated to play a vital role in a range of diseases including obesity and T2DM.^[^ ^45^, ^46^ ^]^ Of note, the crosstalk between AMPK and epigenetic events has been uncovered recently.^[^ ^41^, ^47^ ^]^ Evidence showed that the activity of DNMT1 might be regulated by AMPK.^[^ ^41^ ^]^ Here we fill in the gap by showing that free fatty acid could promote DNMT1 mitochondrial translocation via activation of AMPK, thereby induce *ND6* hypermethylation, mitochondrial dysfunction and IR. Due to the critical role of mitochondrial translocator complexes in assisting the translocation of other proteins into mitochondria, it is noteworthy to investigate whether the components of mitochondrial translocator complexes such as TOMs and TIMs are involved in the process in the future. At the meantime, the underpinning networks of AMPK activation in regulating diverse cellular activities such as DNMT1 mitochondrial translocation, mitochondrial biogenesis, lipid oxidation, glucose metabolism, et al. remain need extensive investigations.

## 4 Conclusion

With the observation of specific *ND6* hypermethylation in both T2DM patients and insulin resistant mice, as well as the discovery of free fatty acid promoting DNMT1 mitochondrial localization via AMPK activation, the present study demonstrates an intriguing network accounting for the onset of insulin resistance related metabolic disorders. Especially, the specific knockdown of *ND6* in mouse liver could sufficiently induce hepatic and systemic IR, further highlighting the importance of ND6 stability in maintaining mitochondrial function and metabolic health. Together with the discovery of DNMT1 mediated epigenetic regulation on *ND6* expression and associated metabolic changes, these findings may shed light on potential therapeutic strategies from a perspective of mitochondrial epigenetics for the prevention and treatment of insulin resistance and related metabolic disorders.

Limitations of the study: Both GTEx project and our analysis of peripheral leukocytes from healthy and insulin resistance subjects reveal the close correlation of *ND6* level with clinical features, yet a direct evaluation of *ND6* expression pattern in liver samples of insulin resistance populations was not provided due to extremely limited access.

## 5 Experimental Section

##### Clinical Sample Collection and Preparation

All investigations were performed in accordance with the guidelines of the 1975 Declaration of Helsinki and the protocol was approved by the ethics committees of the Department of Medicine, Xi'an Jiaotong University and Guizhou Aerospace Hospital (No.2019‐1263). Human blood samples from 39 donors with T2DM and 39 donors with non‐T2DM at the age of 45–65‐year‐old were collected from Guizhou Aerospace Hospital (Zunyi, Guizhou, China). The inclusion standard for recruiting was: the subjects were regarded with fasting glucose ≥7.0 mmol L^−1^ as T2DM, and with fasting glucose <7.0 mmol L^−1^ as non‐T2DM. Also, only the subjects with the genetic background of Chinese Han nationality were selected. The exclusion standard was: subjects were excluded with malignant tumors especially the liver cancer. All the subjects were randomly selected. They were not paired matched.

For sample preparation, fresh fasting venous blood samples were centrifuged immediately (3000 *g*, 10 min) to isolate plasma and whole blood cells. Then, the red blood lysis buffer (Tiangen Biotech Beijing Co,. Ltd, Beijing, China, Cat # RT122) was used to obtain peripheral leukocytes. Both DNA and RNA samples were extracted from the leukocytes and analyzed anonymously.

DNA from the leukocytes was extracted with the QIAamp DNA Mini Kit (Qiagen, Hilden, Germany, Cat # 51 304) according to the standard protocol. Briefly, the leukocytes were lysed in a lysis buffer which contained proteinase K and incubated at 56 °C overnight. Then, the RNase A was used to remove the RNA, then, the ethanol was added into the lysis buffer to precipitate the DNA. Finally, the QIAamp mini spin column was used to further purify the DNA. The quality of the extracted DNA is good with a concentration of 50–100 ng µL^−1^ and an A260/A280 ratio of 1.75–1.85.

RNA from the leukocytes was extracted with TRIzol Reagent (Sigma Aldrich, St. Louis, MO, Cat # T9424) according to the standard protocol. Briefly, the leukocytes were homogenized in TRIzol reagent and centrifuged at 10 000 *g* for 5 min to remove the cell debris. The supernatant was collected and the chloroform‐isopropanol extraction was used to extract and precipitate the RNA. Then the RNA pellets were washed with 70% ethanol, and dissolved in diethyl pyrocarbonate (DEPC) water. To further improve the purity of the extracted RNA, the DNase I was used followed by a RNA purify column (Tiangen Biotech Beijing Co,. Ltd, Beijing, China, Cat # DP431). The quality of the extracted RNA is good with a concentration of 100–200 ng µL^−1^ and an A260/A280 ratio of 1.95–2.05.

##### Animals

All the mice were purchased from Vital River Laboratory Animal Technology Co., Ltd (Beijing, China). The mice were fed in a temperature‐ and light/dark cycle‐controlled animal room with no limitations to food and water. The protocol was approved by the Animal Care and Use Committee of the School of Life Science and Technology, Xi'an Jiaotong University (No.2015‐0018). All investigations were carried out in terms of the United States Public Health Services Guide for the care and use of laboratory animals.

For the insulin resistant mouse models, 5‐week‐old male C57BL/6 mice were randomly grouped into mice fed a normal diet (control, 10% kcal fat content, Cat # D12492, Research Diets, New Brunswick, NJ) and mice fed a high‐fat diet (HFD, 60% kcal fat content, Cat # D12450, Research Diets, New Brunswick, NJ). After 12 weeks of feeding, the mice were fasted overnight and sacrificed; 8‐week‐old male db/db mice with the C57BL/6 genetic background and male C57BL/6 mice were fed a normal diet (10% kcal fat content, Cat # D12492, Research Diets, New Brunswick, NJ) for 9 weeks before fasted overnight and sacrificed.

For the *ND6* siRNA treatment, mouse *ND6* siRNA with agomir modification (3’‐ cholesterol and 2’‐O‐methyl) was synthesized from Shanghai GenePharma Co., Ltd. The two pairs of targeting sequences were 5’‐GGGUUUGUUGGUUGUUUAATT, 5’‐UUAAACAACCAACAAACCCTT (siRNA1) and 5’‐GGCAAUAUAUAGUUGUGCUTT, 5’‐AGCACAACUAUAUAUUGCCTT (siRNA2). An equal mixture of both siRNA1 and siRNA2 with a total amount of 5 mg kg^−1^ was adopted to increase the knockdown efficiency and injected into the 8‐week old male C57BL/6 mice via tail vein. In vivo siRNA delivery was performed every 4 days for a total of 8 days by using Invivofectamine 3.0 transfection reagent (Thermo‐Fisher, Waltham, MA, Cat # IVF3005).

For adenovirus mediated *Dnmt1* overexpression, both mouse wild‐type *Dnmt1* transcript (NM_0 011 99431.1) and variant transcript carrying mitochondrial targeting sequence following previous report ^[^ ^22^ ^]^ were constructed into the pAd/CMV/V5 adenovirus vectors via the ViraPower Adenoviral Expression System (Invitrogen, Carlsbad, CA). Male C57BL/6 mice at the age of 8‐week old were injected with pAd/CMV/V5‐*Dnmt1*, pAd/CMV/V5‐mt*Dnmt1* or pAd/CMV/V5‐NC adenovirus (3 × 10^9^ PFU per 20 g mouse) via the tail vein. After 12 days of injection, the mice were fasted overnight and sacrificed for further biochemical assay. The remaining mice in each group were performed with OGTT and ITT experiments with 3 days interval.

For AAV8 mediated *Dnmt1* knockdown, mouse *Dnmt1* shRNAs were constructed into the pAAV‐U6‐shRNA‐CMV‐EGFP vector and packaged into AAV8. The targeting sequence for *Dnmt1* shRNA was GCTGACACTAAGCTGTTTGTA (TRCN0000039024), which has been confirmed in the previous study.^[^ ^48^ ^]^ AAV infection (3 × 10^11^ vg per 20 g mouse) was performed via tail vein injection of 11‐week old male C57BL/6 mice already fed on HFD for 6 weeks. The HFD feeding was carried for another 6 weeks. The mice in chow group (fed a normal diet) and in HFD + scramble group were administered the same dose of AAV8 containing scramble vector.

For 5‐Aza treatment, 5‐week old male C57BL/6 mice were fed on a HFD for a total of 8 weeks. 5‐Aza (2 mg kg^−1^ day^−1^, 0.4% dimethyl sulfoxide in phosphate‐buffered saline (PBS)) was administrated from week 5 to 8 during HFD feeding through intraperitoneal injection (Maclin, Shanghai, China); 8‐week old db/db mice were treated with 5‐Aza (2 mg kg^−1^ day^−1^) or vehicle through intraperitoneal injection for 4 weeks under a normal diet.

##### Cell Culture and Treatments

Human HepG2, 293T and HeLa cells and mouse 3T3‐L1 and HT22 cells were acquired from the ATCC (Manassas, VA). Cells were cultured in Dulbecco's Modified Eagle's Medium (DMEM) containing 100 U mL^−1^ penicillin, 100 µg mL^−1^ streptomycin, 10% v/v fetal bovine serum and 0.22% sodium bicarbonate at 37 °C in a cell incubator of 5% CO_2_. Transfection was carried out using X‐tremeGENE HP DNA Transfection Reagent (Roche, Basel, Switzerland, Cat # 0 636 623 6001) or Lipofectamine RNAiMAX Transfection Reagent (Invitrogen, Carlsbad, CA, Cat # 13778‐150) in accordance with the manufacturers’ protocols, and the cells were harvested for subsequent analysis after transfection for 48 h. For the PA‐induced cellular IR model, 300 × 10^−6^ m PA (Sigma Aldrich, St. Louis, MO, Cat # P9767) was adopted to treat HepG2 cells for 24 h. HepG2 is a widely used immortalized cell line derived from human liver, and is primarily used in the present study to support the regulating role of DNMT1‐ND6 axle in the development of human insulin resistance in liver. The use of 293T and HeLa cells was intended to show that regulation of ND6 mRNA expression by DNMT1 was a universal mechanism that not uniquely occurred in liver. The use of mouse 3T3‐L1 and HT22 cell was intended to confirm the consistent knockdown efficiency of ND6 siRNAs and their effects on mitochondrial function, which support the mouse in vivo employment of ND6 siRNAs.

##### Plasmids and siRNA

shRNAs were constructed into the pLKO.1‐puro vector. The targeting sequences for human *DNMT1* were 5’‐CCCGAGTATGCGCCCATATTT‐3’ and 5’‐GAGGTTCGCTTATCAACTAAT‐3’. pcDNA3/Myc‐*DNMT1* was obtained from Arthur Riggs as a gift (Addgene plasmid Cat # 36 939, NM_0 01379.4). Human *ND6* esiRNA was purchased from Sigma Aldrich (St. Louis, MO, Cat # EHU101061).

##### Extraction of mtDNA

mtDNA was extracted with a commercial kit in terms of the standard protocol (Biovision, San Francisco, CA, Cat # K280‐50) with some modifications. Briefly, samples were gently lysed by Dounce homogenization, centrifuged at 700 *g* for 10 min to precipitate the nuclei followed by an additional centrifuge at 700 *g* to remove cell debris and nuclei. The supernatant was collected and centrifuged at 10 000 *g* for 30 min to precipitate the mitochondria. The mitochondrial pellets were resuspended in a lysis buffer which contained proteinase K and incubated at 37 °C overnight to isolate mtDNA. To avoid nuclear DNA contamination, Exonuclease V (NEB, Beijing, China, Cat # M0345) was used to digest nuclear DNA according to a previous study.^[^ ^49^ ^]^ Then, the phenol‐chloroform extraction was used to remove the proteins as previously described.^[^ ^29^ ^]^ Finally, the Ampure beads (Beckam Coulter, Indiannapolis, IN, Cat # A63881) were used to further remove short nuclear DNA fragments or residual proteins. The mtDNA was then precipitated with isopropanol, washed with 70% ethanol, and dissolved in ultrapure water for sequencing.

##### Whole‐Genome Bisulfite Sequencing

The whole‐genome bisulfite sequencing (WGBS) was completed in Beijing Genomics Institute (BGI, Beijing, China) as previously described.^[^ ^50^ ^]^ Briefly, the extracted mouse liver mtDNA was fragmented to ≈150 bp using a bioruptor (Covaris S220, Woburn, MA). Then, the fragments of DNA were blunt‐ended and added with dA and adapters at the 3’‐ends followed by a bisulfite conversion step. After treatment of bisulfite, the DNA was amplified using the PCR and sequenced using the Illumina high throughput sequencing system (Illumina 4000, San Diego, CA).

##### WGBS Read Mapping and Methylated‐Cytosine Frequency Analysis

Reads produced from WGBS were mapped to the well‐established mouse mitochondrial reference genome (ensembl V81 Mus_musculus.GRCm38.81, ftp://ftp.ensembl.org/pub/release‐81/gtf/mus_musculus/Mus_musculus.GRCm38.81.gtf.gz). Only one mismatch was acceptable in the “seed” (the high‐quality end of the read, default is 16 bases) while aligning. Other parameters were set as default. Every cytosine site which contained four or more covered reads was adopted to yield the methylated‐cytosine report. Further analysis including the total methylation frequency and averaged methylation frequency of each mitochondrial‐encoded gene were all calculated based on this report.

##### Methylation‐Specific PCR

The extracted mtDNA was fragmented mechanically before treated with the EZ DNA methylation kit (Zymo Research, Orange, CA, #Cat D5005). Detection of DNA methylation was conducted using the real‐time PCR with both the methylated and unmethylated primers which were designed by using the L‐strand of mtDNA *ND6* and D‐loop as templates (Table S2, Supporting Information). The methylated DNA level was expressed as the ratio of methylated DNA to the total DNA level.

##### mtDNA Immunoprecipitation (mtDIP)

mtDIP assay was conducted in terms of the previous study.^[^ ^51^ ^]^ Briefly, the isolated mitochondrial pellets were crosslinked, lysed, and subjected to immunoprecipitation. After washing and elution, DNA from elution buffer was purified and analyzed by real‐time PCR with input DNA used for normalization. The primers adopted in mtDIP are listed in Table S3 (Supporting Information).

##### Confocal Microscopy

HepG2, 293T, and HaLa cells were seeded onto coverslips which coated with poly‐L‐lysine and fixed with 4% paraformaldehyde after transfection for 48 h. The cells were treated with Triton‐X‐100 (Sigma Aldrich, St. Louis, MO, Cat # V900502), washed with PBS and blocked with bovine serum albumin followed by incubated with anti‐mouse‐DNMT1 antibody (Abcam, Cambridge, MA) and anti‐rabbit‐VDAC antibody (Cell Signaling Technology, Danvers, MA). Then the cells were incubated with Alexa Fluor 555‐conjugated anti‐mouse IgG and Alexa Fluor 488‐conjugated anti‐rabbit IgG (Invitrogen, Carlsbad, CA) antibodies. After washing with PBS, the cells were stained with DAPI (Invitrogen, Carlsbad, CA). Images were visualized with a ZEISS LSM700 fluorescence microscope at 63x magnification (Carl Zeiss, Chicago, IL). For detection of *ND6* siRNA mitochondrial localization, mouse *ND6* siRNA with FAM modification was used to transfect 3T3‐L1 cells for 48 h. Then the cells were stained with Mito Tracker Red (Thermo‐Fisher, Waltham, MA, Cat # M7512) to mark mitochondrial morphology. For detection of mitochondrial superoxide, 3T3‐L1 and HT22 cells were transfected with mouse ND6 siRNA for 48 h, and then stained with MitoSox Red (Thermo‐Fisher, Waltham, MA, Cat # M36008).

##### Transmission Electron Microscopy

The small pieces of liver tissues were fixed in 2% glutaraldehyde, rinsed in PBS, post fixed in 1% osmium tetroxide and dehydrated with a graded series of alcohol. Then the samples were embedded and sliced followed by stained with uranyl acetate and lead citrate. Images were observed using a transmission electron microscope (EM902; Carl Zeiss).

##### Isolation of Mitochondria

Mitochondria in cultured cells were isolated according to previous study.^[^ ^52^ ^]^ Briefly, the collected cells were resuspended in hypotonic buffer which contained 10 × 10^−3^ m Tris base, 10 × 10^−3^ m NaCl, and 2.5 × 10^−3^ m MgCl_2_ at a pH of 7.5, homogenized on ice and centrifuged at 1300 *g* for 5 min at 4 °C. The supernatants were collected and centrifuged at 17 000 *g* for 15 min at 4 °C to precipitated the mitochondria, and the mitochondria was lysed with western and immunoprecipitation lysis buffer (Beyotime, Nanjing, China, Cat # P0013) for subsequent western blot assay. Liver mitochondria were isolated in terms of previous study.^[^ ^53^ ^]^ Briefly, liver tissues were washed with PBS followed by transferred in isolation buffer which contained 0.5 × 10^−3^ m EDTA, 10 × 10^−3^ m Tris, and 0.25 m sucrose at a pH of 7.4. The tissues were minced, washed and homogenized in 2.5 vol of isolation buffer. After increasing to an 8x initial vol with isolation buffer, the homogenates were centrifuged at 1000 *g* for 10 min at 4 °C and the supernatant fraction was centrifuged at 10 000 *g* for 10 min at 4 °C. The mitochondrial pellet was rinsed twice with isolation buffer, and the supernatant was collected as the cytosolic fraction. Freshly isolated mitochondria were used immediately for detection of mitochondrial complex activity.

##### Mitochondrial Complex Activity Assay

The activities of NADH‐ubiquinone reductase (complex I), succinate‐coenzyme Q oxidoreductase (complex II), coenzyme Q‐cytochrome c reductase (complex III), cytochrome c oxidase (complex IV), and ATP synthase (complex V) were detected with conventional assays as previously described.^[^ ^2^, ^54^ ^]^

##### Cell Oxygen Consumption Rate (OCR) Assay

HepG2, HeLa, 293T, 3T3‐L1, and HT22 cells were seeded in microplates (Seahorse Bioscience, Billerica, MA) at a density of 1.5–3 × 10^4^ cells mL^−1^. After treating with mitochondrial inhibitors (1 × 10^−6^ m antimycin A, 0.5 × 10^−6^ m FCCP and 1 × 10^−6^ m oligomycin), the OCR was detected using an extracellular flux analyzer (Seahorse Bioscience, Billerica, MA). Basal respiration represents the baseline of the oxygen consumption value before the injection of the mitochondrial inhibitors. Maximal respiration represents the maximum OCR value after injection of FCCP. Spare respiratory capacity is calculated by recording the OCR response to FCCP and dividing the value by the basal respiration. The actual value of OCR was adjusted according to the cellular protein concentration.

##### OGTT and ITT

OGTT was carried out by i.p. injection of glucose at the dose of 1 g kg^−1^ (Sigma Aldrich, St. Louis, MO, Cat # G7021) following a 14 h fasting. ITT was carried out by i.p. injection of insulin at the dose of 0.75 U kg^−1^ (Sigma Aldrich, St. Louis, MO, Cat # I9278) following a 5 h fasting. The levels of glucose in blood samples from the tail vein were detected using test strips (Contour, Bayer, Cat # 7099C).

##### Serum and Liver Chemistry

Serum samples were prepared by centrifugation of the whole blood at 3000 *g* for 10 min. The T‐CHO, TG, cholesterol low‐density lipoprotein (LDL‐c), cholesterol high‐density lipoprotein (HDL‐c), alanine aminotransferase (ALT) activity and aspartate aminotransferase (AST) activity were determined using an automated biochemistry analyzer (Hitachi Ltd., Tokyo, Japan). The level of insulin was detected with a commercial ELISA kit (Invitrogen, Cat # EMINS). Liver tissues were homogenized in 5% NP‐40 buffer and then slowly heated to 100 °C for 5 min. Insoluble materials were eliminated by centrifugation, and the remaining supernatants were diluted 10‐fold with water prior to the detection of the total TG and T‐CHO levels (BioVision, San Francisco, CA).

##### Histological Analysis

Small pieces of liver and adipose tissues were fixed in 4% paraformaldehyde, minced into 3–5 µm of thickness and stained with hematoxylin and eosin. Histological images were observed using an Olympus BX71 microscope.

##### Human Data Bioinformatics Analysis

Differential expression analysis was performed between obese subjects (BMI ≥ 30) and others (BMI < 30) in 49 tissues from the GTEx project (release v8, https://gtexportal.org/home/) for 13 protein coding genes in the mitochondrial genome. A total of 979 subjects were included. The analyses were performed using DESeq2 package implemented in R.

##### RNA Sequencing

The RNA sequencing of mouse liver was completed in Annoroad Gene Technology Corporation (Beijing, China). RNA samples were sequenced using an Illumina platform according to the standard protocol (HiSeq, Illumina, San Diego, CA). Raw data were filtered to obtain clean data. Analysis of both the Kyoto Encyclopedia of Genes and Genomes (KEGG) pathways and the volcano plots were conducted using the clean data.

##### Real‐Time PCR

Total RNA was isolated from human leukocyte, mouse liver tissue or cell samples with TRIzol Reagent (Sigma Aldrich, St. Louis, MO, Cat # T9424) and reverse‐transcribed into cDNA with PrimeScript RT Master Mix (TaKaRa, DaLian, China, Cat # RR047A), followed by semi‐quantitative real‐time PCR with particular primers. For the detection of mtDNA copy number, total DNA was isolated with the QIAamp DNA Mini Kit (Qiagen, Hilden, Germany, Cat # 51 304) and subjected to semi‐quantitative real‐time PCR with mitochondrial D‐loop primers. The 2‐△△Ct method was used to analyze the data, and *18S* rRNA was adopted as a housekeeping gene to normalize the data of both mRNA and DNA. The results are presented as a percentage of the control. The primers adopted in real‐time PCR are listed in Table S4 (Supporting Information).

##### Western Blot

Tissue and cell samples were washed with PBS and homogenized with Western and immunoprecipitation lysis buffer (Beyotime, Nanjing, China, Cat # P0013). The homogenates were centrifuged at 13 000 *g* for 15 min at 4 °C, and the supernatants were collected for subsequent detection of protein concentrations with a BCA Protein Assay kit (Pierce, Rockford, IL, Cat # 23 225). 10–20 µg protein samples were subjected to SDS‐polyacrylamide gel electrophoresis and transferred to nitrocellulose membranes (PerkinElmer Life Science, Boston, MA). After blocking with 5% nonfat milk, the membranes were incubated with primary antibodies followed by horseradish peroxidase‐conjugated secondary antibodies. Images of the western blots were visualized with an ECL Western blotting detection kit (Pierce, Rockford, IL, Cat # 32 209) and quantified by scanning densitometry. The antibodies used were anti‐DNMT1, anti‐DNMT3b and anti‐Histone H1 (Abcam, Cambridge, MA), anti‐DNMT3a, anti‐VDAC, anti‐Insulin Receptor *β* (IR), anti‐p‐IR (Tyr 1150/1151), anti‐Akt, anti‐p‐Akt (Ser 473), anti‐GSK3*α*/*β*, anti‐p‐GSK3*α*/*β* (Ser 21/9), anti‐GAPDH, anti‐*α*‐tubulin, anti‐*β*‐Actin, anti‐p38, anti‐p‐p38 (Thr180/Tyr182), anti‐ERK1/2, anti‐p‐ERK1/2 (Thr 202/204), anti‐AMPK*α*, anti‐p‐AMPK*α* (Thr172) and anti‐GFP (Cell Signaling Technology, Danvers, MA), anti‐JNK, anti‐p‐JNK (Thr183/Tyr185) and anti‐B23 (Santa Cruz, CA), and anti‐MT‐ND6 (Bioss, Beijing, China, Cat # bs‐3955R). Mitochondrial protein carbonyls were measured with a commercial detection kit (Sigma Aldrich, St. Louis, MO, Cat # S7150).

##### Statistical Analysis

Statistical analyses were conducted with two‐tailed unpaired Student's *t* test for two comparisons, or with one‐way ANOVA followed by Bonferroni's post hoc analysis for multiple comparisons. GraphPad Prism 6 was adopted to analyze the data. The results are presented as the means ± SEM. For all analyses in the study, values of *p* < 0.05 were regarded as statistically significant.

## Author Contributions

K.C., W.L., and X.W. contributed equally to this work. J.L. conceived and supervised the study, Z.F., J.L., W.Z., F.G., and J.L. designed the experiments. K.C., W.L., and X.W. performed most experiments with the help of X.Z., S.D, T.Y., X.L., J.X., M.Z., D.Z., Q.M., and M.L., K.C., and Z.F. analyzed the data. K.C., Z.F., and J.L. wrote the manuscript.

## Conflict of Interest

The authors declare no conflict of interest.

## Supporting information

Supporting Information

## Data Availability

The sequencing data were submitted to GEO repository (Hypermethylation of hepatic mitochondrial ND6 provokes systemic insulin resistance, GSE 111996, ID: 200111996).
