# Supplementary material for: Hypermethylation of Hepatic Mitochondrial ND6 Provokes Systemic Insulin Resistance
Source: Adv Sci (Weinh). 2021 May 2;8(11):2004507. doi: 10.1002/advs.202004507 (PMC8188198; doi:10.1002/advs.202004507)
Supplement: Supplementary file 1 — Supporting Information [file ADVS-8-2004507-s001.pdf]

© 2021 Wiley-VCH GmbH

ADVANCED SCIENCE
Open Access

Supporting Information

for *Adv. Sci.,* DOI: 10.1002/advs.202004507

Hypermethylation of hepatic mitochondrial *ND6* provokes systemic insulin resistance

Ke Cao, Weiqiang Lv, Xueqiang Wang, Shanshan Dong, Xuyun Liu, Tielin Yang, Jie Xu, Mengqi Zeng, Xuan Zou, Daina Zhao, Qingqing Ma, Mu Ling, Jiangang Long, Weijin Zang, Feng Gao, Zhihui Feng, Jiankang Liu**

Supporting Information

Hypermethylation of hepatic mitochondrial *ND6* provokes systemic insulin resistance

Ke Cao, Weiqiang Lv, Xueqiang Wang, Shanshan Dong, Xuyun Liu, Tielin Yang, Jie Xu, Mengqi Zeng, Xuan Zou, Daina Zhao, Qingqing Ma, Mu Ling, Jiangang Long, Weijin Zang, Feng Gao, Zhihui Feng, Jiankang Liu**

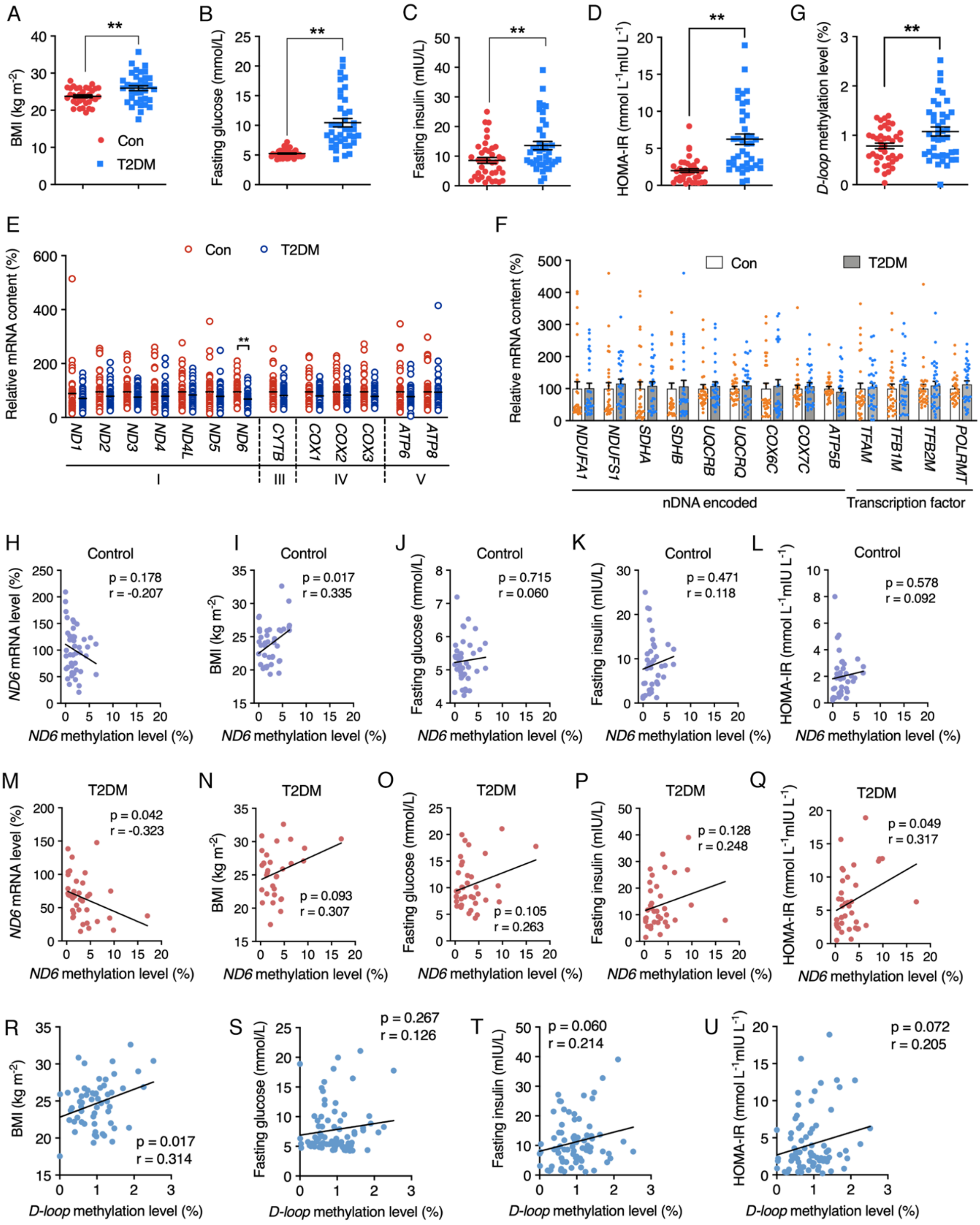

Figure S1 Linear correlations between clinical parameters and *D-loop* methylation level. A-D, BMI, fasting glucose level, fasting insulin level, and HOMA-IR index in T2DM and non-T2DM subjects. **E**, Relative mRNA levels of 13 mtDNA-encoded OXPHOS complex subunits; **F**, nDNA-encoded OXPHOS complex subunits and mtDNA-related transcription factors; **G**, *D-loop* methylation level in human peripheral leukocytes of T2DM and non-T2DM subjects, in **A-G**, *n*=39 for each group. **H-L,** Liner correlations between *ND6* mRNA level, BMI, fasting glucose, fasting insulin, HOMA-IR, and ND6 methylation level in human peripheral leukocytes of non-T2DM subjects, *n*=39; **M-Q,** Liner correlations between *ND6* mRNA level, BMI, fasting glucose, fasting insulin, HOMA-IR, and ND6 methylation level in human peripheral leukocytes of T2DM subjects, *n*=39; **R**, Linear correlations between BMI and *D-loop* methylation level; **S**, fasting glucose and *D-loop* methylation level; **T**, fasting insulin and *D-loop* methylation level; **U**, HOMA-IR index and *D-loop* methylation level in human peripheral leukocytes, *n*=78. Values are mean ± SEM. **p< 0.01.

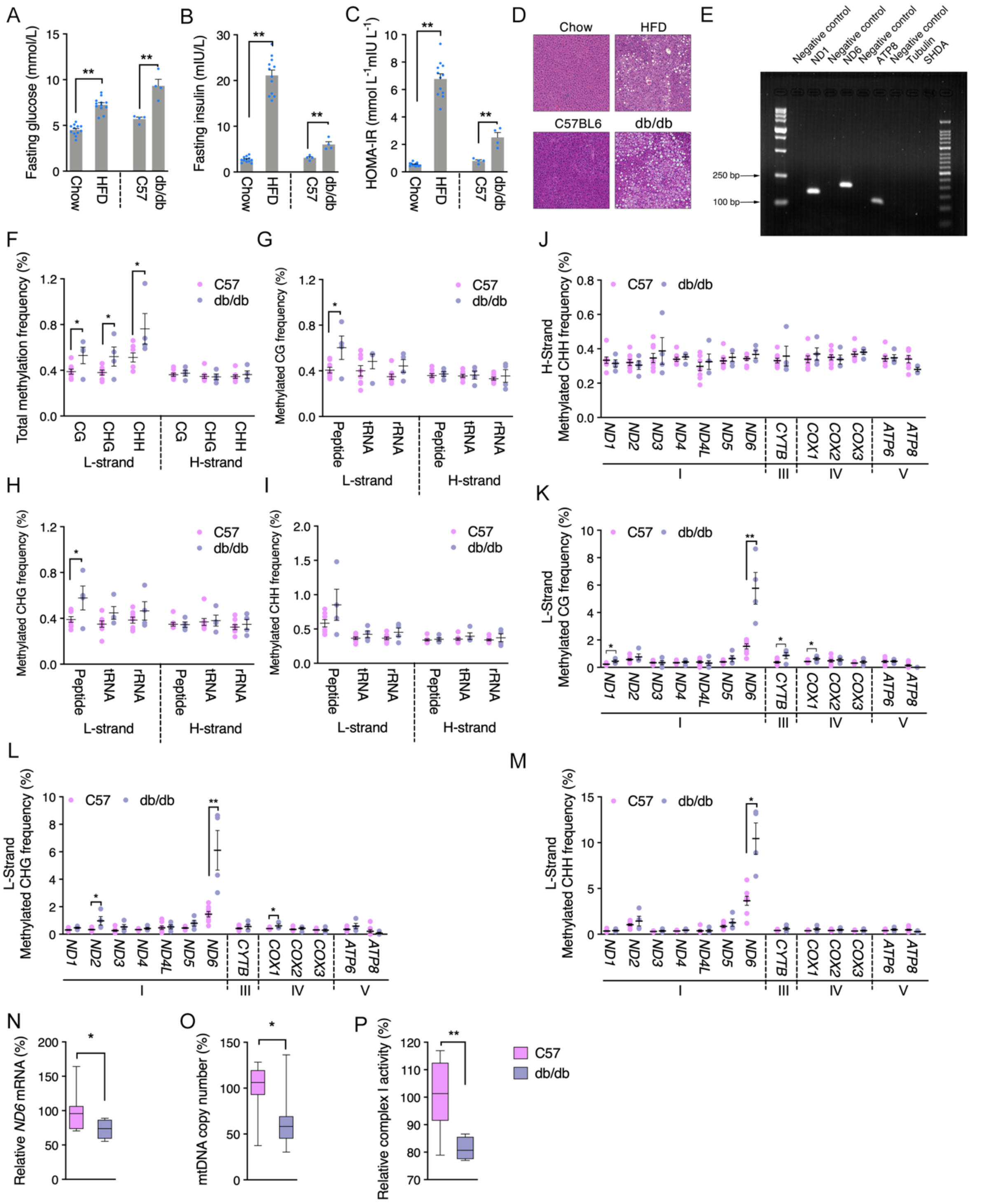

Figure S2 ***ND6*** **is a primary target of mtDNA methylation in the liver of db/db mice. A-C**, Fasting glucose, fasting insulin, and HOMA-IR index in HFD and db/db mice. **D**, HE staining of liver tissues. **E**, Confirmation the purity of extracted mtDNA via PCR with specific mitochondrial and nuclear primers. NC1-4, negative control of *ND1*, *ND6*, *ATP8* and *Tubulin*, respectively. Predicted PCR products were *ND1*, 152 bp; *ND6*, 199 bp; *ATP8*, 113 bp; *Tubulin*, 294 bp; *Sdha*, 247 bp. **F**, Total methylated CG, CHG and CHH frequencies; **G-I**, Methylated CG, CHG, and CHH frequencies of peptides, tRNAs and rRNAs on liver mtDNA. **J-M**, Methylated CHH frequencies on H-strand, methylated CHH, CHG, and CG frequencies on L-strand of 13 oxidative phosphorylation (OXPHOS) complex subunits in the liver of control and

db/db mice. **N**, Relative *ND6* mRNA level; **O**, Relative mtDNA copy number; **P,** Relative complex I activity in the liver of the control and db/db mice. Values are mean ± SEM. *n*=7 for the control and 4 for the HFD group, *p< 0.05, **p< 0.01.

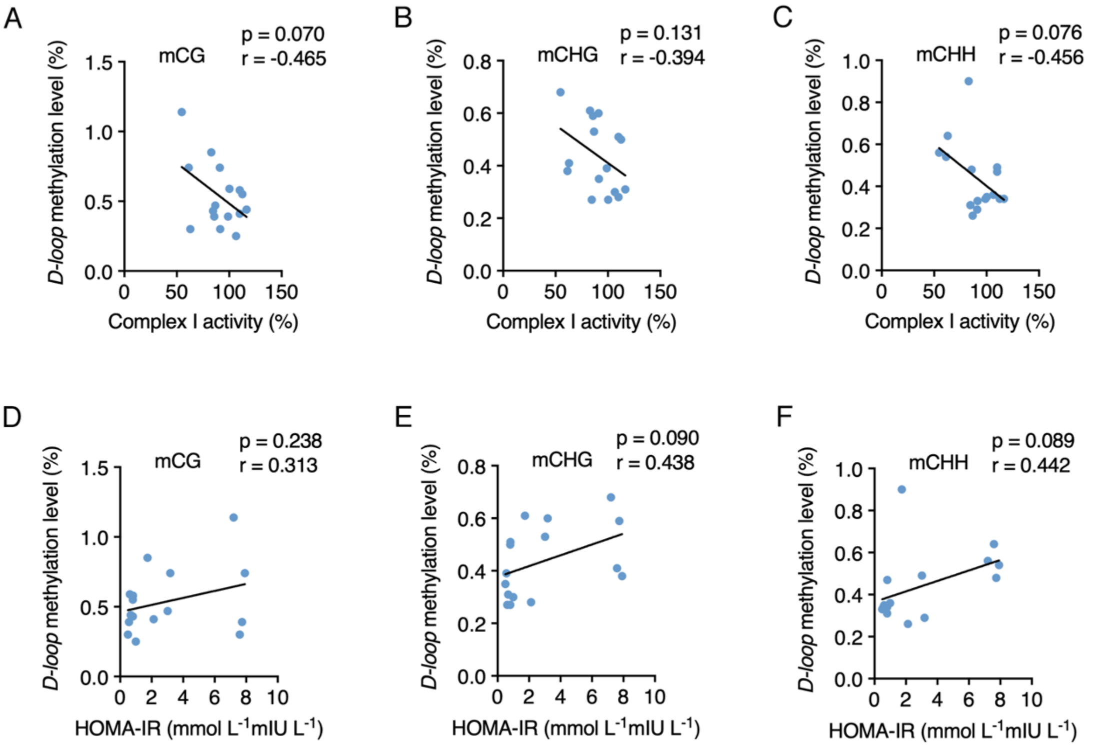

Figure S3 Corralations between D-loop methylation and complex I activity and HOMA-IR. A-C, Liner correlations between *D-Loop* methylated CG, CHG, CHH frequencies and complex I activity in the liver of control and db/db mice; **D-F**, Liner correlations between *D-Loop* methylated CG, CHG, CHH frequencies and HOMA-IR index in the liver of control and db/db mice. N=15.

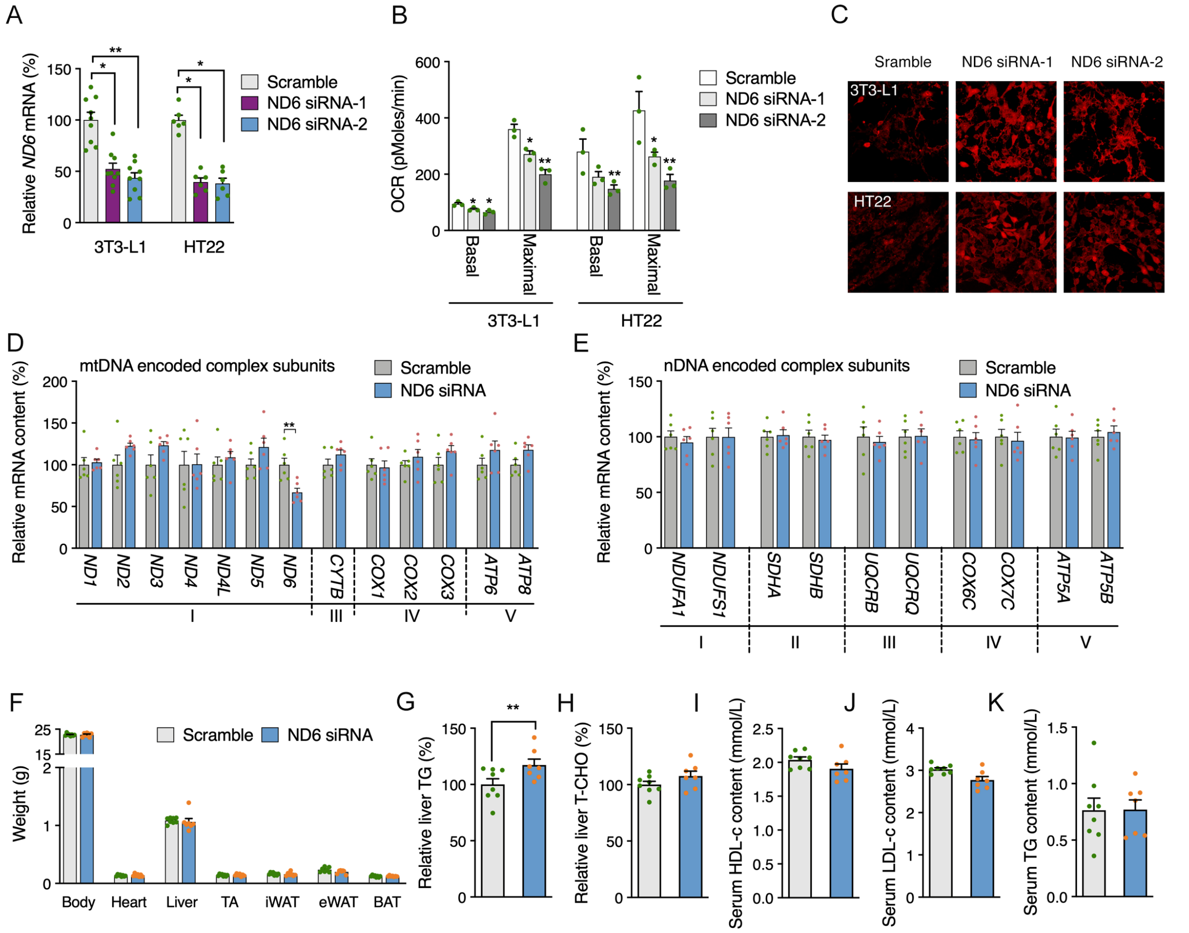

Figure S4 *ND6* knockdown promotes mitochondrial dysfunction in murine cell lines and hepatic lipid accumulation in mice. A, Relative *ND6* mRNA levels; **B**, mitochondrial OCR; **C**, fluorescence of mitochondrial superoxide in 3T3-L1 and HT22 cells transfected with *ND6* siRNA (n=3). **D,** Relative mRNA levels of 13 mtDNA-encoded OXPHOS complex subunits in the liver of ND6 siRNA treated mice (n=6). **E,** Relative mRNA levels of nDNA-encoded OXPHOS complex subunits in the liver of ND6 siRNA treated mice (n=6). **F**, Body and tissue weight; **G**, Relative liver TG; **H**, Relative liver T-CHO; **I**, Serum HDL-c; **J**, serum LDL-c of mice; **K**, Serum TG levels in mice treated with *ND6* siRNA for one week ($n \geq 6$). Values are mean ± SEM. *p< 0.05, **p< 0.01.

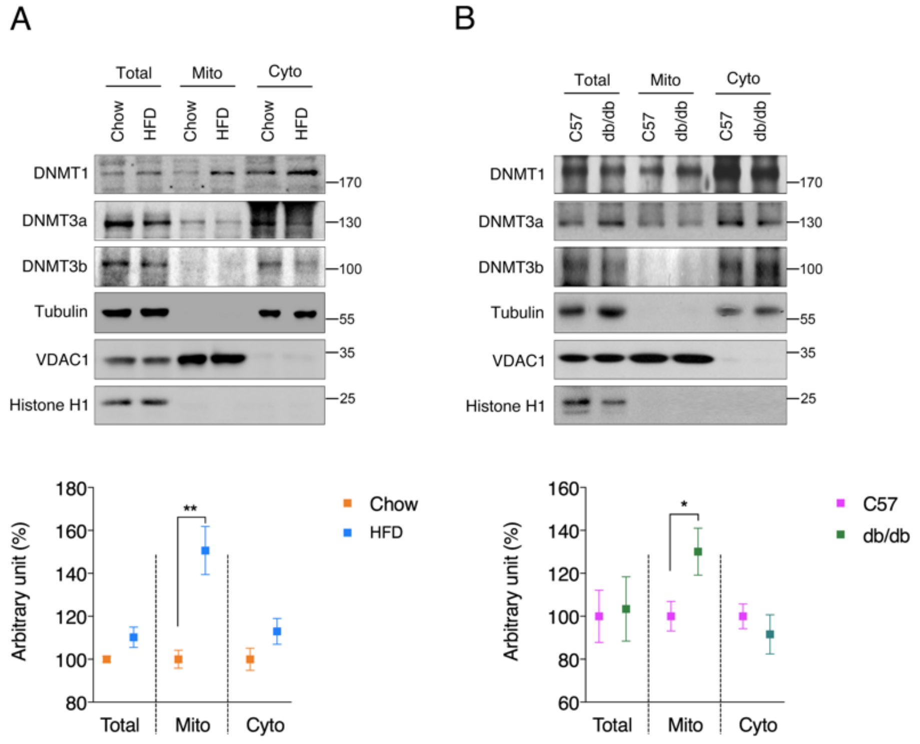

Figure S5 Increased mitochondrial DNMT1 localization in the liver of HFD mice and db/db mice. A-B, Western blot analysis of liver DNMT1, DNMT3a and DNMT3b protein levels in total, mitochondrial and cytosolic fractions of HFD mice and (b) db/db mice. Values are mean ± SEM. *n*=6, *p< 0.05, **p< 0.01.

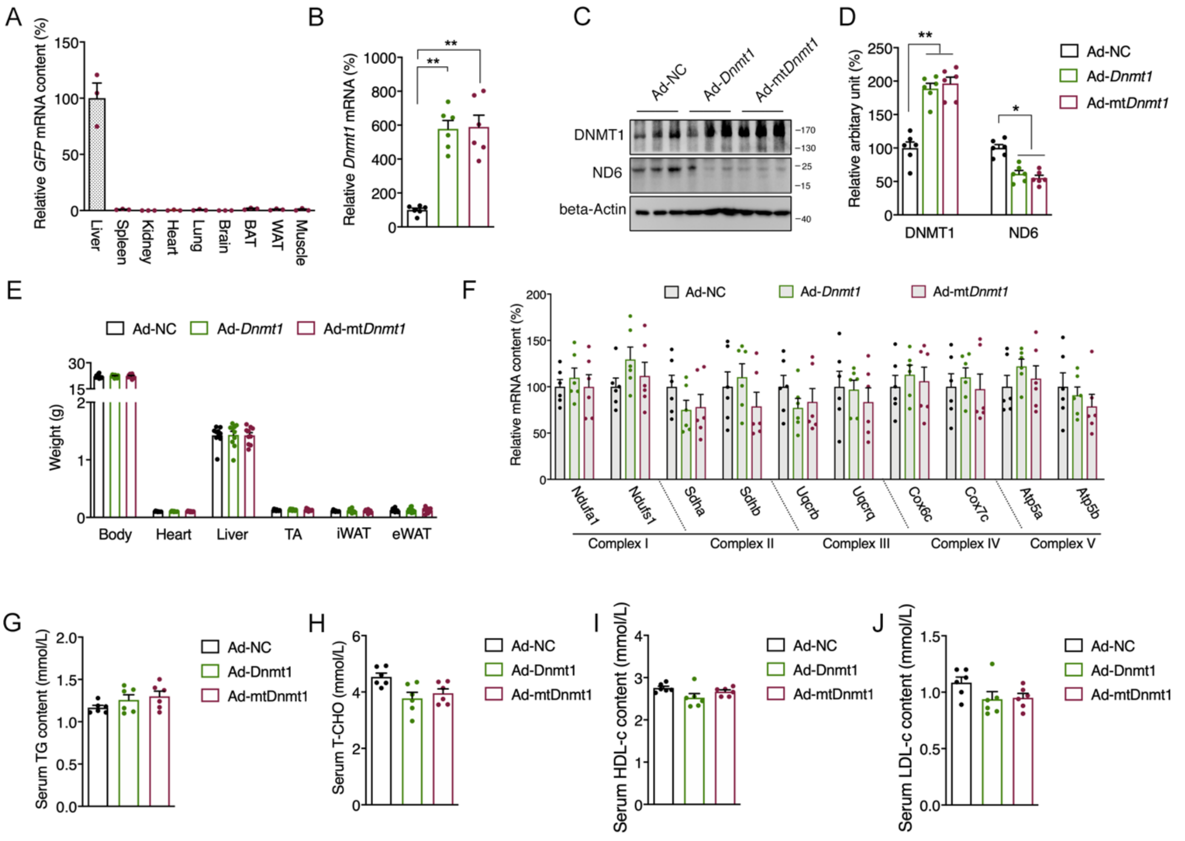

Figure S6 Characterization of mouse adenovirus infection through tail vein injection. A, *GFP* mRNA expression in the tissues of mice infected with pAd/CMV/V5-*GFP* adenovirus via the tail vein injection (*n*=3). **B,** Relative *Dnmt1* mRNA level in the liver of mice infected with pAd/CMV/V5-*Dnmt1*(Ad-*Dnmt1*), pAd/CMV/V5-mt*Dnmt1*(Ad-mt*Dnmt1*) or pAd/CMV/V5-NC (Ad-NC) adenovirus (*n*=6). **C-D,** Protein levels of DNMT1 and ND6 in the liver of mice infected with adenovirus (*n*=6). **E,** Body and tissue weight of mice after *Dnmt1* overexpression (*n*=8). **F,** Relative mRNA level of nDNA-encoded OXPHOS complex subunits (*n*=6). **G,** Serum TG; **H,** Serum T-CHO; **I**, Serum HDL-c; **J**, Serum LDL-c of mice infected with pAd/CMV/V5-*GFP* adenovirus via the tail vein injection (*n*=6). Values are mean ± SEM. *p< 0.05, **p< 0.01.

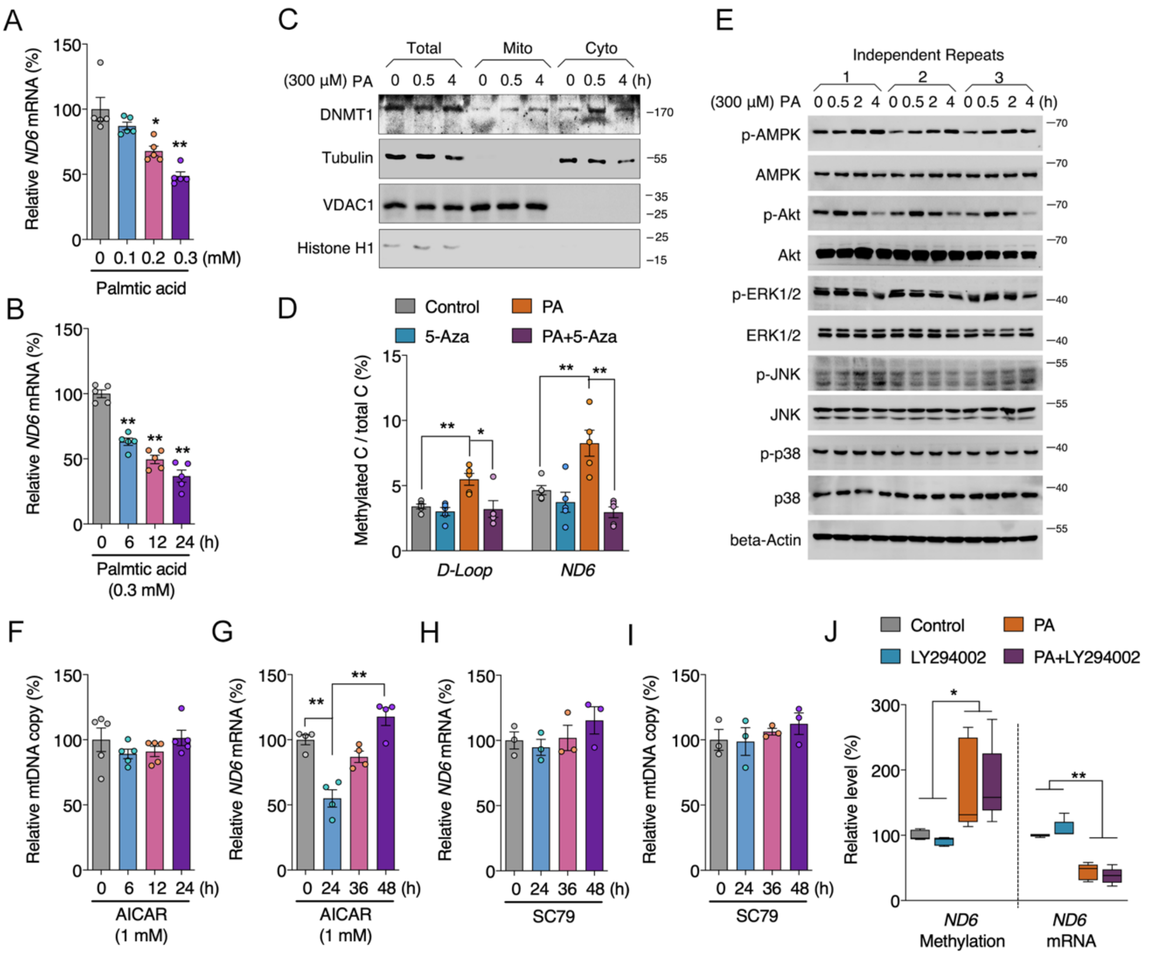

Figure S7 AMPK activation is associated with PA induced *ND6* hypermethylation by DNMT1. A, Relative *ND6* mRNA level in HepG2 cells under dose dependent PA treatment for 24 h (*n*=5). **B,** Relative *ND6* mRNA level in HepG2 cells under time dependent PA treatment at 300 μM (*n*=5). **C,** Western blot analysis of DNMT1 protein subcellular localization in mouse primary hepatocytes under 300 μM PA treatment. **D,** Methylation levels of *D-loop* and *ND6* in PA treated cells with or without 2 μM 5-Aza for 24 h (*n*=5). **E,** Western blot of p-AMPK, AMPK, p-Akt, Akt, p-ERK1/2, ERK1/2, p-JNK, JNK, p-p38 and p38 in HepG2 cells under time dependent PA treatment at 300 μM. **F,** mtDNA copy number in 1 mM AICAR treated cells (*n*=4). **G,** Relative *ND6* mRNA level in cells after prolonged AICAR treatment (*n*=4). **H,** Relative *ND6* mRNA level in HepG2 cells under time dependent 2 μM SC79 treatment (*n*=3). **I,** Relative mtDNA copy number in HepG2 cells under time dependent 2 μM SC79 treatment (*n*=3). **J,** *ND6* methylation and mRNA levels in PA treated cells with or without 10 μM LY294002 for 24 h (*n*=5). Values are mean ± SEM. *p< 0.05, **p< 0.01.

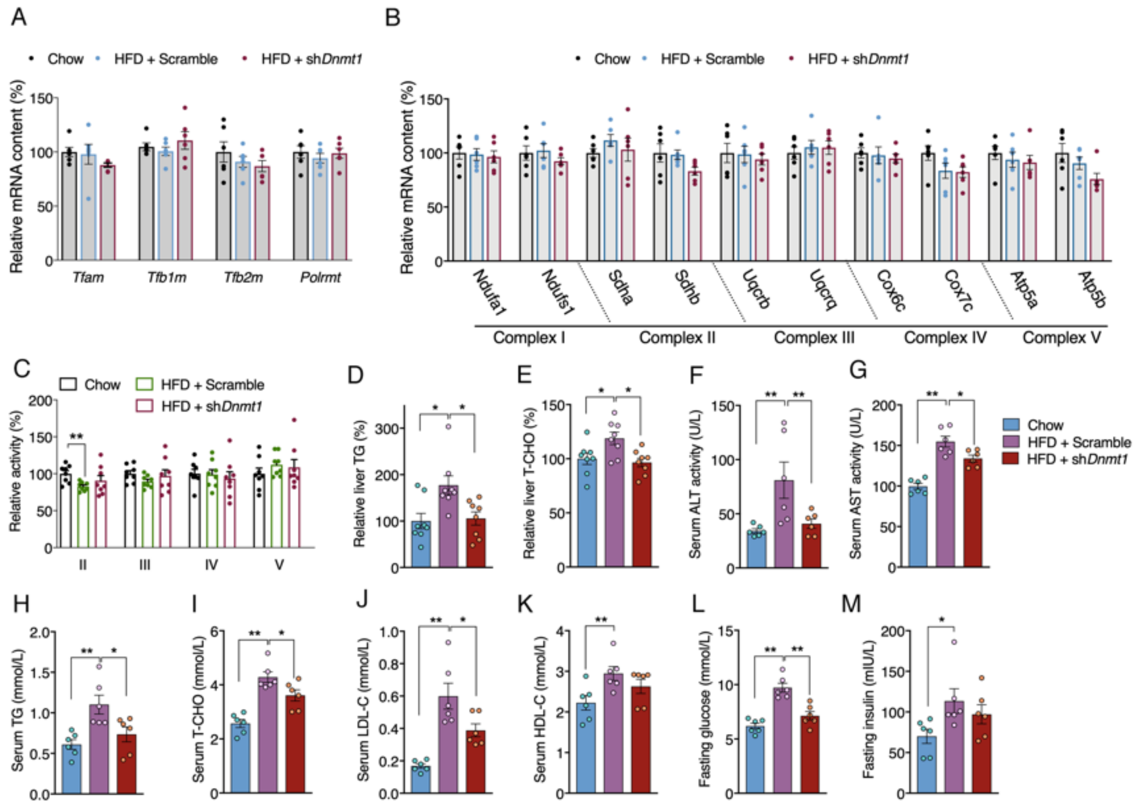

Figure S8 Hepatic knockdown of *Dnmt1* ameliorates metabolic abnormality induced by HFD. A-B, Relative mRNA levels of mtDNA-related transcription factors and nDNA-encoded OXPHOS complex subunits in the liver of mice under diets and AAV intervention (*n*=6). **C,** Relative complex II-V activities in liver (*n*=8). **D-E,** Relative TG and T-CHO level in the liver (*n*=8). **F-K,** Serum ALT activity, AST activity, TG level, T-CHO level, LDL-c level, and HDL-c level in mice (*n*=6). **L-M,** Fasting glucose and fasting insulin level in mice after diets and AAV intervention (*n*=6). Values are mean ± SEM. *p< 0.05, **p< 0.01.

Table S1 Probabilities of DNMT1 isoforms to mitochondria predicted by MitoProt Ⅱ.

Species	Isoform	Length (amino acid)	Probability to mitochondria	Mitochondrial leader peptide
Human	NM_001130823.3 NP_001124295.1 DNMT1 isoform a	1632	47.94%	MPARTAPARV
Human	NM_001318730.2 NP_001305659.1 DNMT1 isoform c	1619	48.33%	MPARTAPARV
Human	NM_001318731.2 NP_001305660.1 DNMT1 isoform d	1511	38.96%	MADANSPPKPLSKPRTPRRS
Human	NM_001379.4 NP_001370.1 DNMT1 isoform b	1616	47.33%	MPARTAPARV
Human	DNMT1 isoform b supplemented with an additional MTS reported by Shock et al. (mtDNMT1)	1717	85.54%	MNECLGHRTHLPANRGAWTS PLLRVGGVCARLAHACSLGM AGSVPSFCTGYRLSPFGTSPPP PRPDWGGRRRLRSSPLPIGFRA KSRG
Human	NM_003201.3 NP_003192.1 TFAM isoform 1 precursor	246	92.26%	MAFLRSMWGVLSALGRSGAE LCTGCGSRLRSPFSFVYLPRWF SSVLASC
Mouse	NM_001199431.1 NP_001186360.2 Dnmt1 isoform 1	1620	36.13%	MPARTAPARV
Mouse	NM_001199432.1 NP_001186361.1 Dnmt1 isoform 3	1501	72.45%	MADSNRSPRSRPKPRGPRRS
Mouse	NM_001199433.1 NP_001186362.1 Dnmt1 isoform 4	1502	72.45%	MADSNRSPRSRPKPRGPRRS
Mouse	NM_001314011.1 NP_001300940.1 Dnmt1 isoform 5	1627	36.42%	MPARTAPARV
Mouse	NM_010066.4 NP_034196.5 Dnmt1 isoform 2	1619	36.13%	MPARTAPARV
Mouse	Dnmt1 isoform 1 supplemented with an additional MTS reported by Shock et al. (mtDnmt1)	1673	91.70%	MRTPFGHSMVFPHSLALCGT CCFRLRRPLPIGFRAREKAGV SFRAVLSSATCKMPARTAPARV

Table S2 Primers used in methylation-specific PCR.

Species	Region	Primer (5'-3')
Human	*D-loop-M*	Forward: CGTTTTTTTTAAATAAGATATTACGA<br>Reverse: AAAAATCAAAAACAAATACTACGAC
Human	*D-loop-U*	Forward: TGTTTTTTTTAAATAAGATATTATGA<br>Reverse: AAAAATCAAAAACAAATACTACAAC

Human	*ND6-M*	Forward: TTTCGTATTAATAGGATTTTTCGA<br>Reverse: AATTATCTTTAAATATACTACAACGAT
Human	*ND6-U*	Forward: TTTTGTATTAATAGGATTTTTTGA<br>Reverse: ATAATTATCTTTAAATATACTACAACAAT
Mouse	*D-loop-M*	Forward: GTTTTTTTAAATAAGATATTTCGA<br>Reverse: TACTATCCTTTCATACCTTAACGAC
Mouse	*D-loop-U*	Forward: GTTTTTTTAAATAAGATATTTTGA<br>Reverse: TACTATCCTTTCATACCTTAACAAC
Mouse	*ND6-M*	Forward: GATATTTTTTAGTAGTTATAGTAGTCGT<br>Reverse: TAATTTTAAAATTTAATAAATCGTT
Mouse	*ND6-U*	Forward: TGGATATTTTTTAGTAGTTATAGTAGTTGT<br>Reverse: TAATAATTTTAAAATTTAATAAATCATT

Table S3 Primers used in mtDIP.

Species	Region	Primer (5'-3')
Human	*D-loop*	Forward: TGCTTGTAAGCATGGGGAGG<br>Reverse: ACATTACTGCCAGCCACCAT
Human	*ND6*	Forward: GGGGTTTGTGGGGTTTTCTTC<br>Reverse: ACCCATATAACCTCCCCCAA

Table S4 Primers used in real-time PCR.

Species	Gene	Primer (5'-3')
Mouse	*ND6*	Forward: CTACCCCAATCCCTCCTT<br>Reverse: GGTTTGGTGGATCGTTTT
Mouse	*Dnmt1*	Forward: GAGGCTGATGATGATGTGTC<br>Reverse: GGAATCATCTGGAATGACCG
Mouse	*Ndufa1*	Forward: ATGTGGTTCGAGATTCTCCC<br>Reverse: GCAACTCGTTTTTCCTTGC
Mouse	*Ndufs1*	Forward: ATCCCATAGGTCAGACAAGC<br>Reverse: CAGTTGTGCGAACATATCCT
Mouse	*Sdha*	Forward: AAGATTACAAAGTGCGGGTC<br>Reverse: TTTCATCAGTAGGAGCGGAT
Mouse	*Sdhb*	Forward: GTTCTCGGCAGAGTCGG

		Reverse: TCCGCACTTATTCAGATCCA
Mouse	*Uqcrb*	Forward: CACTCTCAGGTCAAAATGGC<br>Reverse: ATCTCGCATTAACCCCAGT
Mouse	*Uqcrq*	Forward: GCGTCTATCTTCTGTCCCA<br>Reverse: ACCACTACAAACGGCGG
Mouse	*Cox6c*	Forward: CATTGGCTACCATGAGTTCC<br>Reverse: CAGCCACGCCAAACTTATAG
Mouse	*Cox7c*	Forward: GAGCAAGGTCGTGTAGAAAG<br>Reverse: GAAAATGGCAAATTCTTCCCC
Mouse	*Atp5a1*	Forward: CATTTTGTGCCAGTCGTCC<br>Reverse: ATTTTTGGAGACCAGTCCCG
Mouse	*Atp5b1*	Forward: AATTCCTGTTGGTCCTGAGA<br>Reverse: CCAAAGAGTCCGATTTTCCC
Mouse	*Tfam*	Forward: AGACTACACTGGGAAACCAC<br>Reverse: GTTAGGCTTATAGGGACCCA
Mouse	*Tfb1m*	Forward: CCCAAACCAAAAGTTGATGT<br>Reverse: TTTCCTTCGAAACTGAAACG
Mouse	*Tfb2m*	Forward: GGCATTACTTAAAGCTGGTG<br>Reverse: TTTATAGGAACACCTGCTGAC
Mouse	*Polrmt*	Forward: TTCCCTCAGGAGTTTGTCTG<br>Reverse: TGATAGGGCTGTATGATGGG
Mouse	*18S rRNA*	Forward: GTAACCCGTTGAACCCCATT<br>Reverse: CCATCCAATCGGTAGTAGCG
Mouse	*D-loop*<br>(for DNA)	Forward: AGGCATGAAAGGACAGCA<br>Reverse: TTGGCATTAAGAGGAGGG
Mouse	*18S rRNA*<br>(for DNA)	Forward: GAGAAACGGCTACCACATCC<br>Reverse: CACCAGACTTGCCCTCCA
Human	*ND1*	Forward: TTCGCCCTATTCTTCATAGC<br>Reverse: GGAGGTTAGAAGTAGGGTCT
Human	*ND2*	Forward: ACACCCTTAATTCCATCCAC<br>Reverse: GAGATAGGTAGGAGTAGCGT
Human	*ND3*	Forward: AGAAAAATCCACCCCTTACG<br>Reverse: CATGGTAGGGGTAAAAGGAG
Human	*ND4*	Forward: CTCTCTGTGCTAGTAACCAC

		Reverse: ATGGGGGATAGGTGTATGAA
Human	*ND4L*	Forward: CACACCTCATATCCTCCCTA<br>Reverse: GCATTGGAGTAGGTTTAGGT
Human	*ND5*	Forward: CCTATTCGCAGGATTTCTCA<br>Reverse: AATGTGCATAGTGGGGATTT
Human	*ND6*	Forward: CAACCATCATTCCCCCTAAA<br>Reverse: GTGGGTTTAGTAATGGGGTT
Human	*CYTB*	Forward: AAACTTACTATCCGCCATCC<br>Reverse: GTAAGGGTGGAAGGTGATTT
Human	*COX1*	Forward: CCTAATCACAGCAGTCCTAC<br>Reverse: ATGGGAGATTATTCCGAAGC
Human	*COX2*	Forward: AAACCGTCTGAACTATCCTG<br>Reverse: AATGGGGGAAGTATGTAGGA
Human	*COX3*	Forward: CGCTAAATCCCCTAGAAGTC<br>Reverse: AGCCAATAATGACGTGAAGT
Human	*ATP6*	Forward: GATCCCCACCTCCAAATATC<br>Reverse: TAAGAGATCAGGTTCGTCCT
Human	*ATP8*	Forward: AACACAAACTACCACCTACC<br>Reverse: CAATGAATGAAGCGAACAGA
Human	*NDUFA1*	Forward: ACCCAGAAGTAGGGTTTTGG<br>Reverse: TTCTCCAAACCCTTTGACAC
Human	*NDUFS1*	Forward: CAAGGATTCTAGTCCCTCCG<br>Reverse: CTTAACATATTGCTTCTCCCCG
Human	*SDHA*	Forward: AACATGGAGGAGGACAACTG<br>Reverse: CATATCGCAGAGACCTTCCA
Human	*SDHB*	Forward: TTGTTCCCGATTTGAGCAAC<br>Reverse: TCACGCTCTTCTATGGACTG
Human	*UQCRB*	Forward: TGGTCAAAATGGCTGGTAAG<br>Reverse: CTCGCATTAACCCCAGTTTA
Human	*UQCRQ*	Forward: TCAGCTACAGCTTGTCACC<br>Reverse: AAACACTACAAACTGCGGC
Human	*COX6C*	Forward: GCTTTGTATAAGTTTCGTGTGG<br>Reverse: AACTGAGAAAGACTTACTGTCC
Human	*COX7C*	Forward: GATCTGCATTTGCTACACCC

		Reverse: GGCTGCACCTCTTAAAATGC
Human	*ATP5B1*	Forward: CCTGTTGGTCCTGAGACTTT<br>Reverse: CCAAAAAGCCCAATTTTGCC
Human	*TFAM*	Forward: CCAAGAAGCTAAGGGTGATT<br>Reverse: TGTTTCTTTATTGTGCGACG
Human	*TFB1M*	Forward: AAGTTGTTAAGACTGCAAGC<br>Reverse: TTTCAACCACCAGAAGTTCA
Human	*TFB2M*	Forward: TGGATGGAAAACTACGAGTG<br>Reverse: GGAACTGCTTCTATTCCCAA
Human	*POLRMT*	Forward: AAGGTCAAGCAAATAGGAGG<br>Reverse: GTCAGGCCCTTCCTGTA
Human	*18S rRNA*	Forward: GTAACCCGTTGAACCCCATT<br>Reverse: CCATCCAATCGGTAGTAGCG
Human	*D-loop*<br>(for DNA)	Forward: CAGTACCTAACAAACCCACA<br>Reverse: GAGGTCGTAAACCCTATTGT
Human	*18S rRNA*<br>(for DNA)	Forward: TGGTGAGCTGCGAGAATAG<br>Reverse: TTTTATGGTAATAACGCGGC
